# Computational Prediction and Experimental Realization of Earth-Abundant Transparent Conducting Oxide Ga-Doped ZnSb_2_O_6_

**DOI:** 10.1021/acsenergylett.2c01961

**Published:** 2022-10-10

**Authors:** Adam J. Jackson, Benjamin J. Parrett, Joe Willis, Alex M. Ganose, W. W. Winnie Leung, Yuhan Liu, Benjamin A. D. Williamson, Timur K. Kim, Moritz Hoesch, Larissa S. I. Veiga, Raman Kalra, Jens Neu, Charles A. Schmuttenmaer, Tien-Lin Lee, Anna Regoutz, Tung-Chun Lee, Tim D. Veal, Robert G. Palgrave, Robin Perry, David O. Scanlon

**Affiliations:** × Scientific Computing Department, Science and Technology Facilities Council, Rutherford Appleton Laboratory, Harwell Science and Innovation Campus, Didcot, OxfordshireOX11 0QX, U.K.; ⊗ London Centre for Nanotechnology and Department of Physics and Astronomy, University College London, Gordon Street, LondonWC1E 6BT, U.K.; ¶ Diamond Light Source Ltd., Harwell Science and Innovation Campus, Didcot, OxfordshireOX11 0DE, U.K.; § Department of Chemistry, University College London, 20 Gordon Street, LondonWC1H 0AJ, U.K.; ∥ Thomas Young Centre, University College London, Gower Street, LondonWC1E 6BT, U.K.; ⊥ Department of Materials, Imperial College London, Exhibition Road, LondonSW7 2AZ, U.K.; # Department of Materials Science and Engineering, Norwegian University of Science and Technology (NTNU), Trondheim7491, Norway; @ Department of Chemistry, Yale University, New Haven, Connecticut06520-8107, United States; △ Institute of Materials Discovery, University College London, Malet Place, LondonWC1E 7JE, U.K.; ∇ Department of Physics and Stephenson Institute for Renewable Energy, University of Liverpool, LiverpoolL69 7ZF, U.K.; □ ISIS Pulsed Neutron and Muon Source, Rutherford Appleton Laboratory, Harwell Science and Innovation Campus, Didcot, OxfordshireOX11 0QX, U.K.

## Abstract

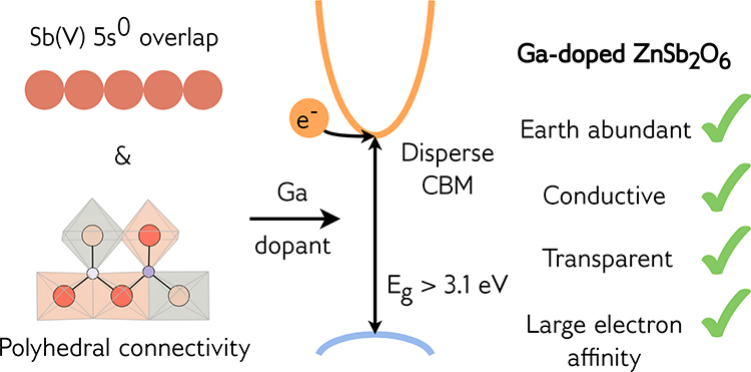

Transparent conducting oxides have become ubiquitous in modern optoelectronics. However, the number of oxides that are transparent to visible light and have the metallic-like conductivity necessary for applications is limited to a handful of systems that have been known for the past 40 years. In this work, we use hybrid density functional theory and defect chemistry analysis to demonstrate that tri-rutile zinc antimonate, ZnSb_2_O_6_, is an ideal transparent conducting oxide and to identify gallium as the optimal dopant to yield high conductivity and transparency. To validate our computational predictions, we have synthesized both powder samples and single crystals of Ga-doped ZnSb_2_O_6_ which conclusively show behavior consistent with a degenerate transparent conducting oxide. This study demonstrates the possibility of a family of Sb(V)-containing oxides for transparent conducting oxide and power electronics applications.

Transparent conducting oxides (TCOs) are an essential component of modern photovoltaic and display screen technologies. Thin-film Sn-doped In_2_O_3_ (ITO) displays the superior optoelectronic properties among the industrially used TCOs; it has been reported to possess resistivities as low as 8 × 10^–5^ Ω·cm, mobilities that exceed 50 cm^2^ V^–1^ s^–1^, and carrier concentrations on the order of 1 × 10^21^ cm^–3^, all while retaining over 90% transparency to visible light.^1^ The more earth-abundant TCOs, such as F-doped SnO_2_ (FTO)^2^ or Al-doped ZnO (AZO),^3^ display mobilities and conductivities below those of ITO, which limits their application in display screen technologies. However, it is undesirable to continue to use ITO for large-area applications such as in photovoltaics, despite recent improvements in the efficiency of indium-based TCOs via innovative doping (Mo and Ce),^4−6^ due to the expense and scarcity of indium. Therefore, there is a drive to try to increase the performance of the known earth-abundant TCOs^7,8^ or, more unusually, to discover new TCOs. The last “new” materials demonstrated to be TCOs were the thin-film correlated metals SrVO_3_ and CaVO_3_ in 2015 and single-crystal La-doped BaSnO_3_ in 2012.^9,10^

In terms of materials design, the common trend in the majority of the effective n-type TCOs is the presence of post-transition-metal cations with the electronic structure (*n* – 1)*d*^10^*ns*^0^*np*^0^. In these materials, the valence *s* orbitals of the cation hybridize with antibonding oxygen 2*p* states, yielding conduction bands with low electron effective masses.^11^ Indeed, the majority of the cations in the industrially relevant TCOs are limited to groups 12, 13, and 14 of the periodic table.

In an early investigation of ternary oxides, Shannon et al. noted that edge-sharing Cd^2+^, In^3+^, and Sn^4+^ octahedra were a feature of common transparent conductors.^12^ Mizoguchi and Woodward built on this in 2004, investigating the necessity for edge-sharing octahedral connectivity when designing n-type TCOs.^13^ They found that edge-sharing is not a prerequisite, and corner-sharing can also provide excellent dispersion of the conduction band, such as in BaSnO_3_.^10^ Interestingly, they identified some ternary oxides containing Sb(V) and Bi(V) which displayed reasonable curvature of the conduction band minimum (CBM),^13^ including tri-rutile zinc antimonate, ZnSb_2_O_6_. It should be noted that group 15 cations in their highest oxidation states possess the same (*n* – 1)*d*^10^*ns*^0^*np*^0^ electronic structure as the cations in the common, successful TCOs.

In 2014, a computational screening study from Hautier et al. also proposed ZnSb_2_O_6_ as a potential transparent conductor, in particular noting its low electron effective mass and earth-abundant, nontoxic composition.^14^ Several other stibnates and germanates were highlighted, but none was followed up with a defect chemistry analysis showing n-type dopability. On the few occasions that ZnSb_2_O_6_ has been synthesized experimentally, transparent conducting behavior has not been observed. Kikuchi et al. investigated it as a TCO and thermoelectric in 2005,^15^ but it was only produced as a powder, and little data on the optoelectronic properties were published. Thick films of crystalline ZnSb_2_O_6_ have been deposited for gas-sensing applications via dip-coating and vapor-phase oxidation methods,^16,17^ although no assessment of their transparent conducting potential has been reported. Spin-coating of a nanoparticle precursor followed by high-temperature annealing was found to result in transparent insulating ZnSb_2_O_6_ thin films, which deteriorated in terms of both transparency and crystallinity upon F-doping.^18^ Finally, Li et al. briefly investigated ZnSb_2_O_6_ as a potential anode for Li battery technology.^19^ Synthesis of high-quality thin films or single crystals of ZnSb_2_O_6_ has not been realized, and its full potential as a transparent conductor has yet to be assessed.

In this work, we investigated the crystal and electronic structure of ZnSb_2_O_6_ with hybrid density functional theory, and validated this description with quasi-particle self-consistent GW theory (Green’s function, *G*, with a screened Coulomb interaction, *W*). A full intrinsic defect analysis was performed that showed that, when nominally undoped, ZnSb_2_O_6_ does not fulfill the Mott criterion for metallic-like conductivity. We then considered three extrinsic dopants, and demonstrated that Ga is the optimum electron donor in ZnSb_2_O_6_. Using this knowledge, we successfully grew powder and single-crystal samples of Ga-doped ZnSb_2_O_6_, which displayed a low absorption coefficient (88 cm^–1^) in the visible range, electron mobility up to 49 cm^2^ V^–1^ s^–1^, conductivity up to 1890 S cm^–1^, carrier concentrations on the order of 2 × 10^20^ cm^–3^, and a Haacke figure of merit comparable with that of polycrystalline ITO. We calculated the electron affinity of ZnSb_2_O_6_ to be nearly 1 eV larger than that of currently available transparent electrodes, offering much needed diversity when considering the band alignment of potential contact layers in devices. Ga-doped ZnSb_2_O_6_ displays all the indicators of a high-performance transparent conductor, with the added bonus of being comprised of earth-abundant elements: Zn and Sb are several orders of magnitude cheaper per kilogram than In, while Ga is required in only very small amounts.^20^ This study stands as an important proof-of-concept for Sb(V)-based TCO design, and invites the development of thin-film deposition to further investigate the optoelectronic properties of ZnSb_2_O_6_ and to prepare it for use in devices and commercial applications.

## Crystal Structure

**Figure 1:**
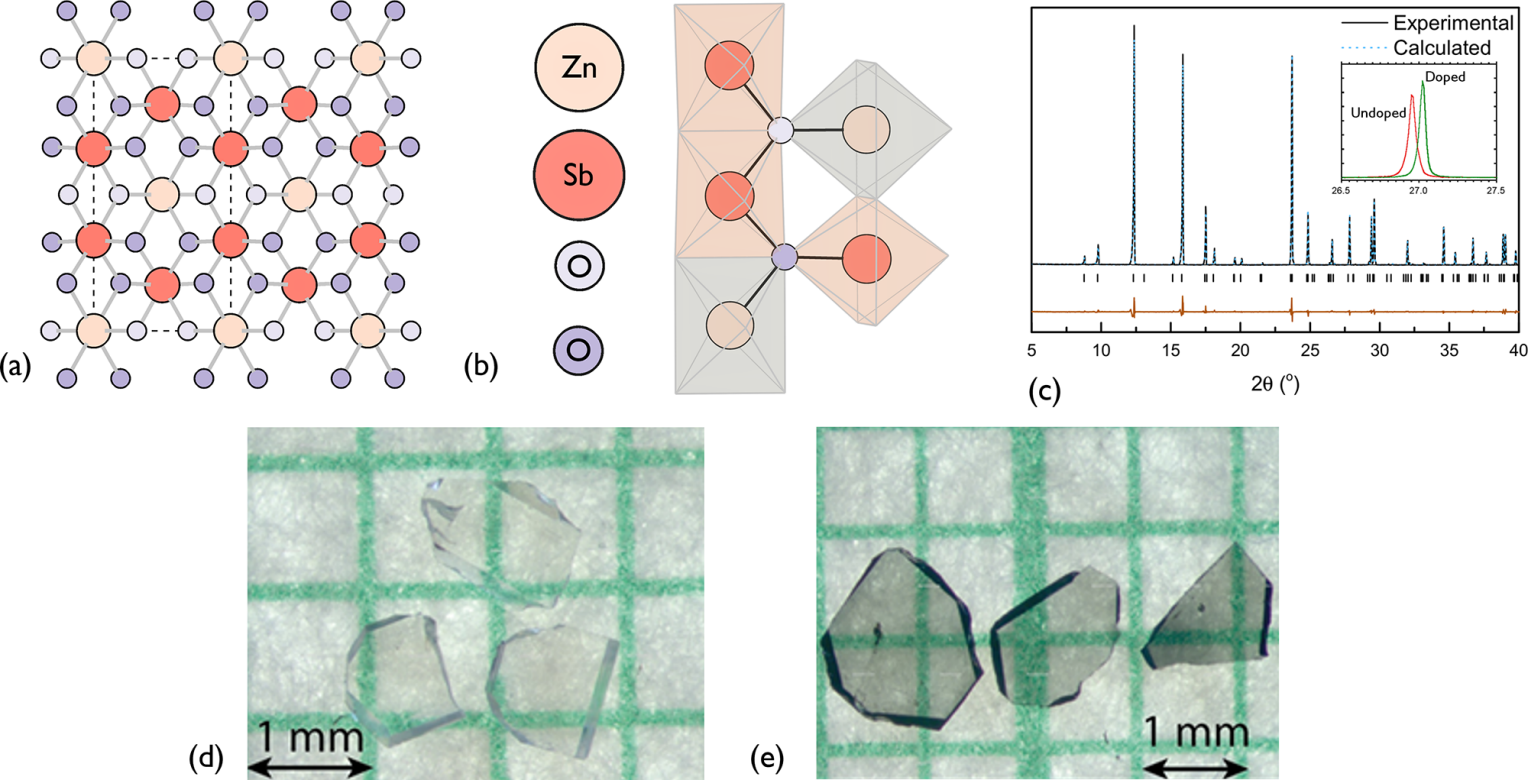
(a) View along the *a*-axis of the crystal structure of ZnSb_2_O_6_. (b) Octahedral connectivity in ZnSb_2_O_6_, viewed from a slight offset of the *a*-axis, which is edge-sharing in the *c*-direction and corner-sharing in the *a*- and *b*-directions. (c) Powder and simulated X-ray diffraction patterns for ZnSb_2_O_6_. The dashed line indicates Rietveld refinement for *P*4_2_/*mnm* tri-rutile structure; the difference between fit and data is shown below the peak positions. The inset shows the typical shift in an XRD peak upon Ga-doping. (d) Undoped ZnSb_2_O_6_ single crystals and (e) Ga-doped ZnSb_2_O_6_ single crystals. All crystal structures were visualized using VESTA.^22^

ZnSb_2_O_6_ crystallizes in a tri-rutile structure, belonging to the *P*4_2_/*mnm* space group, as shown in Figure 1a. The structure consists of ZnO_6_ and SbO_6_ edge-sharing octahedra in the order ZnO_6_–SbO_6_–SbO_6_ along the *c*-axis, with corner-sharing octahedra present throughout the *a–b* planes (Figure 1a,b). The unit cell is tetragonal, and a summary of the fundamental parameters is provided in Table S1 for a range of exchange correlation functionals and experiments. The powder X-ray diffraction (XRD) results show an excellent fit to the *P*4_2_/*mnm* space group, as can be seen in Figure 1c, and the calculated PBE0 lattice parameters are in good agreement with room-temperature XRD results. We note the PBEsol lattice parameters are slightly overestimated, which is typical of the generalized gradient approximation (GGA) implementation in DFT.^21^

Doping with Ga causes a systematic shift in the Bragg peaks to higher angles compared to those of undoped samples, illustrated in the inset of Figure 1c, indicating a shrinkage in cell size. This observation is consistent with substituting Ga with Zn in an octahedral environment, as Ga has a smaller ionic radius (0.62 Å) compared to Zn (0.74 Å).^23^ The unit cell volumes shift from 201.78 Å^3^ to 201.48 Å^3^ after 6% Ga doping, extracted by Rietveld refinement using the GSAS-II software.^24^ These observations are consistent with a solid solution of gallium replacing zinc in the tri-rutile structure, with no detectable phase separation. Optical images of our transparent undoped and Ga-doped single crystals are shown in Figure 1d,e, approximately 1 mm^2^ in area and polished to a thickness of around 150 μm.

## Electronic Structure

**Figure 2:**
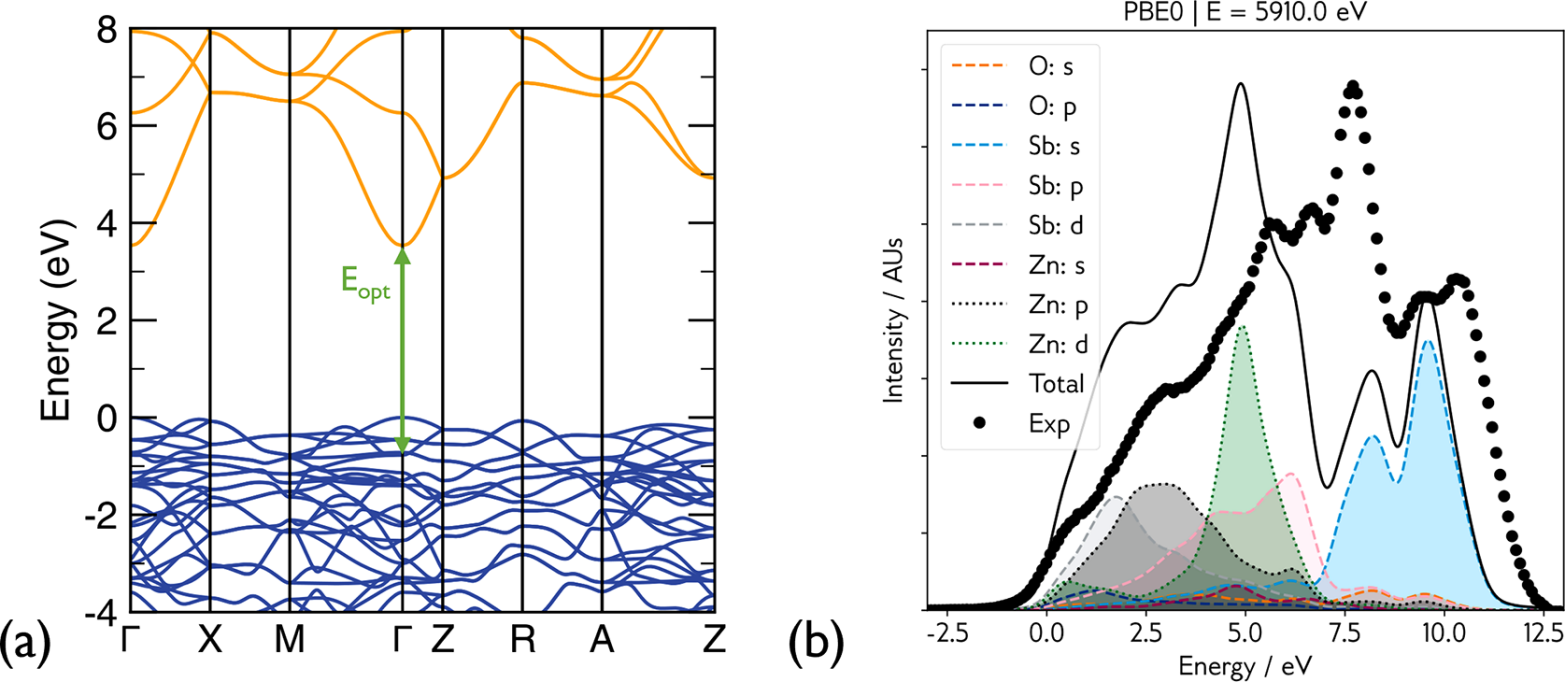
(a) Electronic band structure of ZnSb_2_O_6_ computed using the PBE0 functional. (b) Simulated valence band spectra using the PBE0 functional, weighted by the photoionization cross sections for a photon energy of 5.91 keV, and broadened by a Gaussian of 0.6 eV and a Lorentzian of 0.2 eV. Plotted in black is the HAXPES experimental valence band spectrum of a nominally undoped single crystal, collected at a photon energy of 5.91 keV. Both spectra are normalized to the peak maxima.

We begin with a discussion of the electronic structure, arguably the most decisive indicator of a prospective transparent conductor. The electronic band structure of ZnSb_2_O_6_ was calculated using the PBE0 functional and is displayed in Figure 2a. A direct band gap of 3.54 eV at Γ is observed, with relatively high dispersion at the CBM. The electron effective mass in the Γ→X and Γ→M directions is 0.27*m*_e_, and improves further along Γ→Z (in the *c*-direction) to 0.22*m*_e_. The high dispersion originates from the broad overlap of Sb 5*s* orbitals, which are the main contributors to the CBM density of states, with Zn and O *s* states. Qualitatively, the conduction band shape is in good agreement with previous GGA-DFT calculations, while the hybrid functional corrects for the systematic underestimation of the band gap,^14^ which we note is competitive with state-of-the-art TCOs In_2_O_3_, SnO_2_, ZnO, and BaSnO_3_.^2,7,8,25^Figure S6 shows the band structure computed with hQSGW theory, which shows a small (3%) decrease in the direct band gap to 3.41 eV, with the electron effective masses unchanged. Ultimately, the PBE0 description is sufficient, accurately describing the nature of the band gap compared to the next level of theory, and is used subsequently for defect calculations. The transition from valence band maximum (VBM) to CBM is symmetry forbidden, and the first allowed transition occurs from states around 0.7 eV below the VBM (similar to the case in In_2_O_3_),^26^ as denoted by the green arrow in Figure 2a. This is discussed in greater detail in the Optical Properties section.

Simulated and experimental photoelectron spectra of the valence band of undoped ZnSb_2_O_6_ are shown in Figure 2b, plotted using Galore.^27,28^ The simulated spectrum was obtained from the PBE0 density-of-states calculation, where the orbital contributions were weighted with tabulated photoionization cross sections and broadened with Guassian and Lorentzian functions to reproduce the experimental line shapes. The spectra were approximately aligned to the Fermi level. The key valence band features in Figure 2b are in agreement: an initial onset mainly comprised of Zn 3*d*, Sb 4*d*, and O 2*p* states followed by a sharp peak assigned to Zn 3*d* states. However, the position of this peak is under-bound by approximately 3 eV. The under-binding of transition metal *d* states with hybrid DFT is well documented and has been observed in other well-known TCOs, such as the Zn 3*d* states in ZnO and Sn 4*d* states in SnO_2_.^29,30^

## Defect Chemistry

While the electronic structure of ZnSb_2_O_6_ is a promising indicator of high TCO performance, it is the defect chemistry that will ultimately control the electrical properties of the system. Degenerate conductivity is achieved when the charge carrier concentration exceeds the Mott criterion:^32−35^
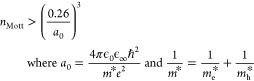
1which for ZnSb_2_O_6_ is 2.6 × 10^18^ cm^–3^, where *a*_0_ is the effective Bohr radius (1.89 × 10^–9^ m), ϵ_0_ is the calculated static dielectric constant (7.74), and m* is the reduced effective mass (1.97 × 10^–31^ kg).

**Figure 3:**
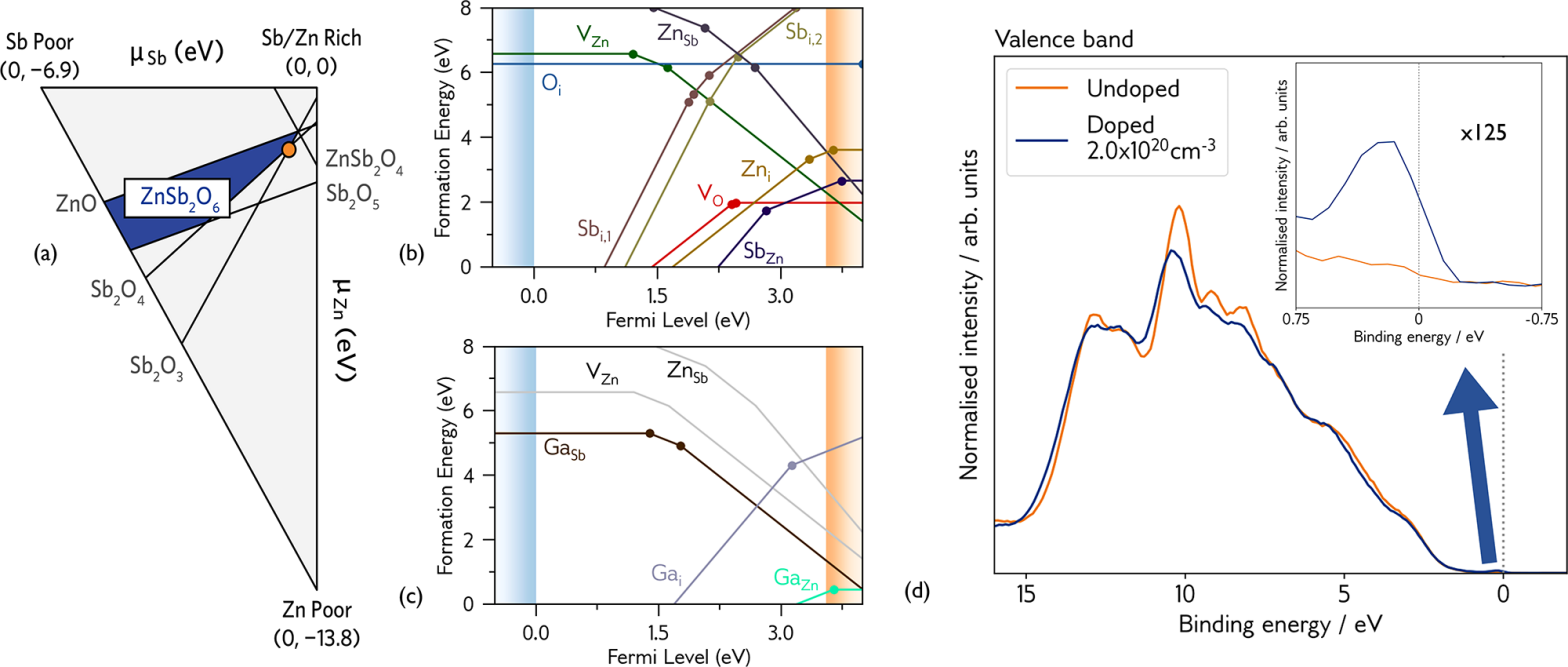
(a) Thermodynamic stability region of ZnSb_2_O_6_, calculated using CPLAP.^31^ The orange marker denotes the most n-type growth conditions, the chemical potential limits at which the defect formation energies in this work are reported. (b) Intrinsic defect transition level diagram of ZnSb_2_O_6_. (c) Extrinsic defect transition level diagram of ZnSb_2_O_6_. (d) Experimental HAXPES valence band spectra of undoped and Ga-doped ZnSb_2_O_6_, normalized to area under the curve. The inset shows a ×125 magnification at the Fermi level, denoted by a dotted gray line. For transition level diagrams, blue and orange shaded regions denote the valence and conduction bands, respectively. Each colored line represents a different defect, and the gradient of that line denotes the charge state. Filled circles represent transition levels, where two charge states are in thermodynamic equilibrium. Calculated and plotted using AIDE. For V_O_, O_i_, F_O_, Zn_i_, and Ga_i_, only the lower energy of the two non-equivalent defect sites is plotted. The gray lines in (c) show the native p-type acceptor defects V_Zn_ and Zn_Sb_.

We first identify the thermodynamic stability region (blue) of ZnSb_2_O_6_ with respect to its competing phases in Figure 3a. This gives an insight into which synthesis conditions are most appropriate for growing n-type ZnSb_2_O_6_ (denoted by the orange circle), and provides the chemical potentials required for calculating the defect formation energies under those synthesis conditions (equation S1). The transition level diagram for intrinsic defects, namely zinc, antimony, and oxygen vacancies (V_Zn_, V_Sb_, and V_O_), cation substitutions (Zn_Sb_ and Sb_Zn_), and various interstitial sites (Zn_i_, Sb_i_, and O_i_), is calculated and displayed in Figure 3b for the most n-type chemical potential limits, corresponding to an oxygen-poor, metal-rich environment.

The intrinsic defect chemistry does not support degenerate n-type behavior. V_O_ acts as a deep donor defect, in line with the behavior observed in established TCOs such as In_2_O_3_, SnO_2_, and ZnO (CdO being the notable exception),^5,8,36−38^ and is discussed in greater detail in the SI. The Sb_Zn_ substitution is the next lowest energy species, but it is charge-compensated by V_Zn_ (see Figure S5) just below the CBM, pinning the Fermi level in the gap. We predict the position of the Fermi level and charge carrier concentrations through a self-consistent Fermi level (SCFL) analysis. The synthesis temperature of our ZnSb_2_O_6_ single crystals is ∼1400 K, and by fixing the defect concentrations present at this temperature and recalculating the SCFL at room temperature, we can predict room-temperature experimental charge carrier concentrations. Undoped ZnSb_2_O_6_ is predicted to have 3.1 × 10^16^ cm^–3^ charger carriers (significantly below the Mott criterion), with the SCFL 0.13 eV below the conduction band edge, precluding undoped ZnSb_2_O_6_ from metallic-like conductivity. From experiment, we measure 5.0 × 10^17^ cm^–3^ carriers in undoped crystals, around an order of magnitude more than predicted, and low conductivity (around 2 S/cm). The slight deviation in carrier concentration measured in the crystals is likely due to adventitious H-doping during synthesis, as well as trace amounts of other impurities that could contribute electrons (such as Cl from the carrier gas), but is in qualitative agreement with the SCFL analysis—i.e., when nominally undoped, ZnSb_2_O_6_ does not display metallic conductivity.

Next, we considered a strategy for electron donation in ZnSb_2_O_6_. Knowing that the Sb *s* states mainly comprise the conduction pathway, we primarily targeted the Zn and O sites for substitution to leave the conduction band unperturbed. Ruling out some of the larger 3+ cations, as they would cause large lattice disruption, Al, Ga (for Zn substitution), and F (for O substitution) were selected as potential dopants for driving the Fermi level into the conduction band and realizing degenerate conductivity.

**Figure 4:**
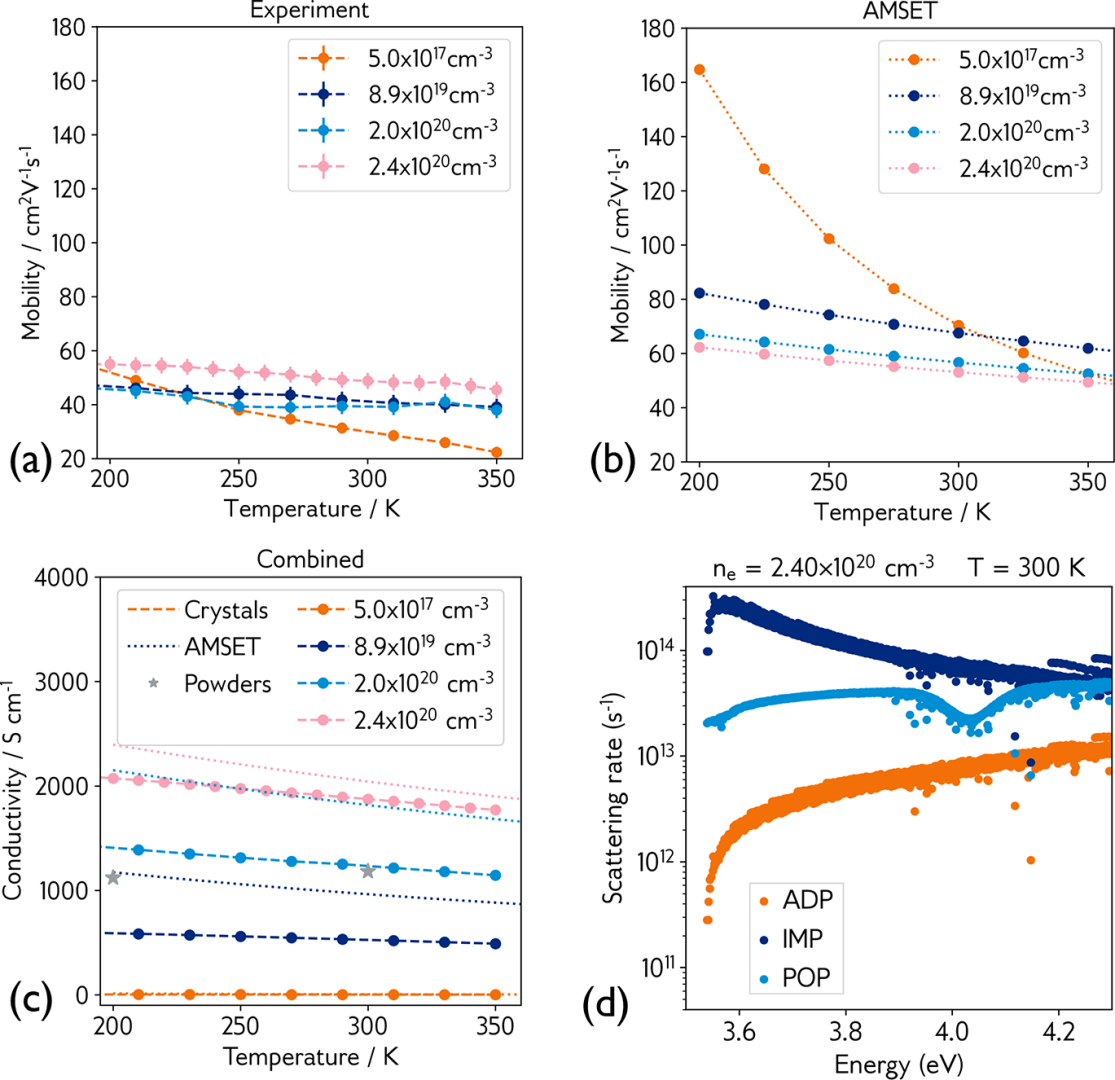
(a) Experimental mobility of undoped and Ga-doped single-crystal ZnSb_2_O_6_. (b) Simulated (using AMSET) mobility of ZnSb_2_O_6_ at carrier concentrations measured from experiment. (c) Single-crystal and simulated conductivity over a range of carrier concentrations, with THz spectroscopy data for an 8% Zn_1–*x*_Ga_*x*_Sb_2_O_6_ solid solution plotted as gray stars. (d) Calculated room-temperature scattering rates at 2.4 × 10^20^ cm^–3^. Abbreviations: ADP, acoustic deformation potential scattering; IMP, ionized impurity scattering; and POP, polar optical phonon scattering. Moving from low to high carrier concentrations causes a switch in dominant scattering from POP to IMP.

The transition level diagram for Ga-doping is shown in Figure 3c, while the Al and F data is presented in Figure S8. We find that Ga_Zn_ and Al_Zn_ are low-energy donors, with formation energies of 0.45 eV and 0.58 eV in their neutral charge states, respectively. In both cases, the dopant interstitial defects are rather high in energy (around 5 eV at the CBM), and are charge-compensated by their respective dopant substitutions onto the Sb site. F_O_ anion substitutions have higher formation energies of 1.38 eV and 1.46 eV for the inequivalent oxygen sites, while the F interstitials do not donate electrons to the conduction band. Crucially, we find that the native p-type defects, V_Zn_ and Zn_Sb_, are too high in energy to charge-compensate Ga_Zn_, Al_Zn_, and F_O_. We complete the same SCFL analysis as before for each case, and find that Ga has a predicted room-temperature charge carrier concentration of 3.4 × 10^19^ cm^–3^ and a SCFL of 3.69 eV (0.15 eV above the CBM), thereby predicting degenerate conductivity. For Al and F, the predicted charge carrier concentrations are 3.2 × 10^19^ cm^–3^ and 7.2 × 10^18^ cm^–3^, with the SCFL sitting above the CBM in both cases. However, Al-doping of ZnSb_2_O_6_ was unsuccessful experimentally, with various Al precursors not responding well to the CVT growth method, and F-doping was not attempted due to the lower predicted achievable carrier concentration, so Ga emerged as the optimal dopant. Experimentally, we record carrier concentrations of 8.9 × 10^19^ cm^–3^, 2.0 × 10^20^ cm^–3^, and 2.4 × 10^20^ cm^–3^ in three single crystals when doped with increasing Ga content. The presence of adventitious H is the most likely origin of discrepancy, but again we find qualitative agreement with the SCFL analysis. Conductivity rises with increasing carrier concentration and Ga-doping content by several orders of magnitude in the single crystals to 526 S cm^–1^, 1230 S cm^–1^, and 1890 S cm^–1^, respectively, and a similar trend is observed in the powders (conductivity plotted in Figures 4c, S1, and S2), competitive with established TCOs.

Through hard X-ray photoelectron spectroscopy (HAXPES), we find further evidence to support this description of the defect chemistry of ZnSb_2_O_6_. At high photon energies (approaching 6 keV), we can exploit the greater photoionization cross-section of Sb 5*s* states, which from the density-of-states calculation we expect make up the CBM, allowing us to observe any filled conduction band states (more information on the HAXPES results can be found in the SI). Figure 3d shows the HAXPES valence band spectra for undoped and Ga-doped ZnSb_2_O_6_ aligned to the Fermi level of the doped sample at 0 eV binding energy, with an expanded view (×125 magnification) provided in the inset between binding energies of 0.75 eV and −0.75 eV. In the undoped sample, there is no photoemission at a binding energy of 0 eV, implying that the conduction band states are unfilled, while the doped sample shows conduction band emission with a typical Fermi–Dirac-like distribution. This is strong evidence of degenerate filling of the conduction band upon Ga-doping, supporting the prediction of metallic-like conductivity from our defect calculations.

## Charge Transport Properties

Charge transport properties are important metrics in assessing the performance of prospective TCOs. Specifically, it is desirable for a TCO to possess high electron mobility in order to maximize conductivity. Figure 4a shows the experimental electron mobility of undoped and Ga-doped ZnSb_2_O_6_ single crystals over the temperature range 200 to 350 K, where we observed an impressive room-temperature mobility of 49 cm^2^ V^–1^ s^–1^ at the maximum doping level (2.4 × 10^20^ cm^–3^), resulting in a conductivity of 1890 S cm^–1^. Our THz domain spectroscopy (TDS) results on powder samples also demonstrate metallic-like conductivity, with a conductivity of 1100 S cm^–1^ achieved at room temperature for Zn_0.92_Ga_0.08_Sb_2_O_6_ (gray stars in Figure 4; the full THz conductivity data set is shown in Figures S1 and S2). Our single crystals display electronic properties that are competitive with those of thin films of ITO, FTO, and AZO, which typically display electron mobilities of 40 cm^2^ V^–1^ s^–1^ to 60 cm^2^ V^–1^ s^–1^ and conductivity on the order of 1 × 10^4^ S cm^–1^.^2,5,7^ In such films, mobility is often limited by ionized impurity scattering, despite the presence of grain boundaries. With suitable deposition conditions and an optimized doping level (sufficiently raising the Fermi level above the conduction band), efficient electron tunneling through grain boundaries can passivate scattering from such defects.^39^ Polycrystalline ZnSb_2_O_6_ films may display reduced electron mobility compared to our single crystals due to grain boundary scattering, but with an optimized deposition process this mechanism could be suppressed as in the other successful doped TCOs.

To further understand the origin of the high mobility in ZnSb_2_O_6_, we performed charge transport calculations using the AMSET package.^40^ This allowed us to calculate the limits to intrinsic mobility from various scattering mechanisms including polar optical phonons (POP), acoustic deformation potentials (ADP), and ionized impurities (IMP). We found that at low carrier concentrations, corresponding to the nominally undoped sample, polar optical phonon scattering dominates, while at higher concentrations the limiting scattering mechanism switches to ionized impurity scattering. This is demonstrated in Figure 4b, where the mobility of the undoped sample displays the strong temperature dependence typically associated with a system dominated by POP scattering, while at high carrier concentrations the mobility becomes largely temperature independent, indicative of IMP-based scattering. We note that in ZnSb_2_O_6_, ADP scattering is largely unimportant, remaining unchanged and at a significantly lower rate than the other mechanisms, regardless of temperature and carrier concentration. In Figure 4d, we explicitly plot the scattering rates at room temperature and high carrier concentration, clearly showing ionized impurity scattering as the dominant scattering mechanism.

There are some discrepancies between the experimental observations and simulations. First, our calculations predict that the nominally undoped material should exhibit a very high electron mobility of around 70 cm^2^ V^–1^ s^–1^ at room temperature and display a strong temperature dependence. However, our undoped crystal shows the lowest mobility and only a weak temperature dependence. The trend of mobility with increased carrier concentration is in fact unclear across the whole batch of samples. Possible causes of these discrepancies include non-uniform distribution of the dopant during the chemical vapor transport (CVT) growth process, other unintentional impurities in the samples (perhaps Ca, as observed in the hard X-ray survey spectrum, Figure S3a) that could have detrimental effects on the mobility, or directional dependence effects during measurement of the single crystals—the mobility of ZnSb_2_O_6_ has reasonable anisotropy, as shown in Figure S7. One other important consideration is that these crystals are very small, only 1 mm^2^, so there is a reasonable error in the measurement. Synthesis of larger single crystals with a refined growth technique could result in improved mobility approaching the value predicted by AMSET; a comparison between calculated and experimental mobility for 24 binary, ternary, and quaternary compounds can be found in ref (40), and is generally reflective of the maturity of the synthesis route. It is also possible that scattering mechanisms not included within the AMSET implementation, such as nonpolar optical phonon scattering, act to lower the experimental mobility and contribute to the discrepancy. Nevertheless, the qualitative agreement between theory and experiment in this study is promising and demonstrates the feasibility and realization of Ga-doped ZnSb_2_O_6_ as a transparent conducting oxide competitive with industry standard materials.

## Optical Properties

Tauc analysis suggests an optical band gap for undoped ZnSb_2_O_6_ of 3.38 eV, which increases to 3.56 eV upon Ga-doping. This widening of the band gap upon Ga-doping is characteristic of the Moss–Burstein shift that is common among the degenerately doped TCOs. The Tauc-derived band gap for the undoped sample is in remarkably good agreement with the predicted fundamental gap of 3.41 eV from the hQSGW calculation.

**Figure 5:**
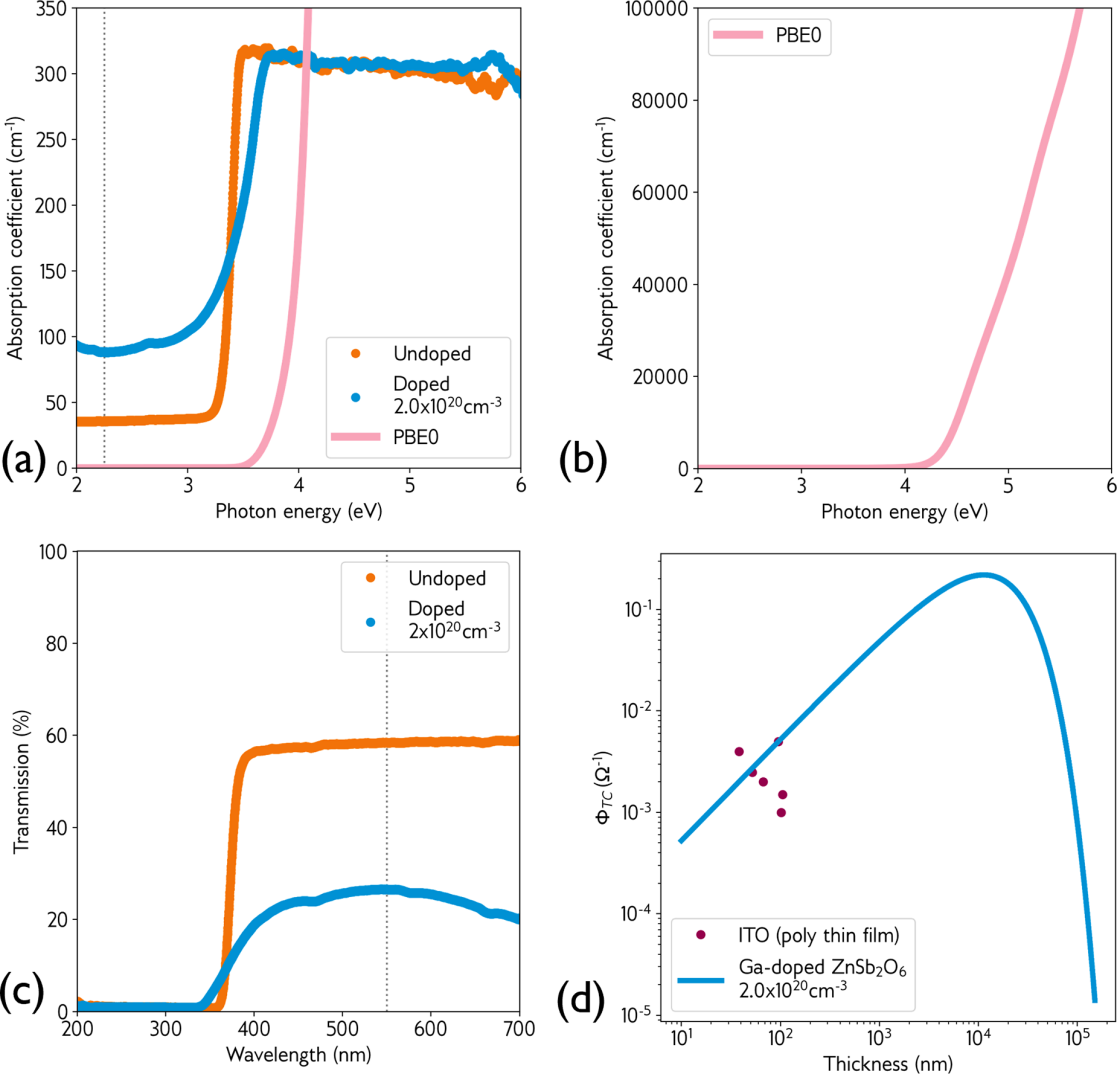
(a) Experimental absorption coefficient of undoped and Ga-doped single crystals, with the calculated PBE0 absorption coefficient (pink) plotted in the same range. (b) Extended plot of the calculated PBE0 absorption coefficient, revealing the much larger value of the optical band gap. (c) Experimental transmission of undoped and Ga-doped single crystals across the visible range. Dotted gray line shows 550 nm on absorption and transmission plot. (d) Haacke figure of merit data at transmittance wavelength of 550 nm, using the doped single-crystal optical data and extracted to different thicknesses, with comparison to data for polycrystalline ITO.^41^

In Figure 5a, the absorption coefficient of undoped and Ga-doped ZnSb_2_O_6_ is plotted over the visible range, along with the calculated PBE0 value. We note that the measured absorption coefficient is below 100 cm^–1^ in both undoped and doped samples at a wavelength of 550 nm, corresponding to the dotted gray line. Further investigation of the computed absorption spectrum revealed a much stronger (several orders of magnitude) absorption onset at around 4.2 eV, shown in Figure 5b. This suggests that the fundamental transition at Γ between the VBM and CBM is forbidden in ZnSb_2_O_6_, and that the actual optical band gap could be up to 0.7 eV larger. Experimentally, the thickness of our single crystals prevents us from probing beyond the initial photon absorption peak, causing the plateau at around 300 cm^–1^. It is known that if sample thickness is too large, transmission can be blocked by a relatively low absorption coefficient beyond the initial onset, as observed in GeSe. In this material, an indirect band gap of around 1.0 eV and absorption coefficient of 150 cm^–1^ are measured in single crystals, and transmission is prevented beyond this energy, while a direct gap of 1.3 eV can be measured in thin films.^42^ Our current calculations predict a large optical band gap of around 4.2 eV, but deposition of thin-film ZnSb_2_O_6_ is required to ratify the true nature and magnitude of the band gap.

The optical transparency of our 150 μm single crystals can be seen in Figure 1d,e and in the transmission intensity plot in Figure 5c. The undoped crystal is transparent and clear, while the Ga-doped crystal displays a blue tint. The corresponding transmission intensities for the single crystals at a wavelength of 550 nm are 58% and 27%, respectively, denoted by the dotted gray line. Clearly, doping has a detrimental effect on the transparency, which has also been observed in the literature when attempting to dope thin-film ZnSb_2_O_6_ with F.^18^ The origin of this decrease in transmission is uncertain—we do not observe any mid-gap states from HAXPES that could signify electrons trapped on defect sites in the band gap. The concentration of V_O_ is not predicted to rise with Fermi level modulation. In contrast, the concentration of Sb_Zn_ is predicted to decrease—this suggests that neither of these defects is responsible for any loss in transmission. It is possible that transitions from the filled conduction band states to the next-available empty band could cause a mild coloration, as has been investigated in other TCOs.^43^ Despite this, moving from a thickness of 150 μm to 150 nm by application of the Beer–Lambert law to the 550 nm transmission data for the doped crystal, the transmission is predicted to exceed 99%, suggesting that, at commercially relevant thicknesses, the optical transparency of ZnSb_2_O_6_ could in fact be superior to that of industry standard ITO.

Finally, we derive the Haacke figure of merit (FOM) for the same Ga-doped crystal, plotted in Figure 5d. This indicates an extremely high FOM at micron thicknesses (approximately 0.2 Ω^–1^), and values competitive with those of industry standard ITO at thicknesses approaching 100 nm. The development of thin-film ZnSb_2_O_6_ will allow a more direct comparison of both optical transmission and FOM, rather than using the predicted thin-film transmission intensities.

## Band Alignment

**Figure 6:**
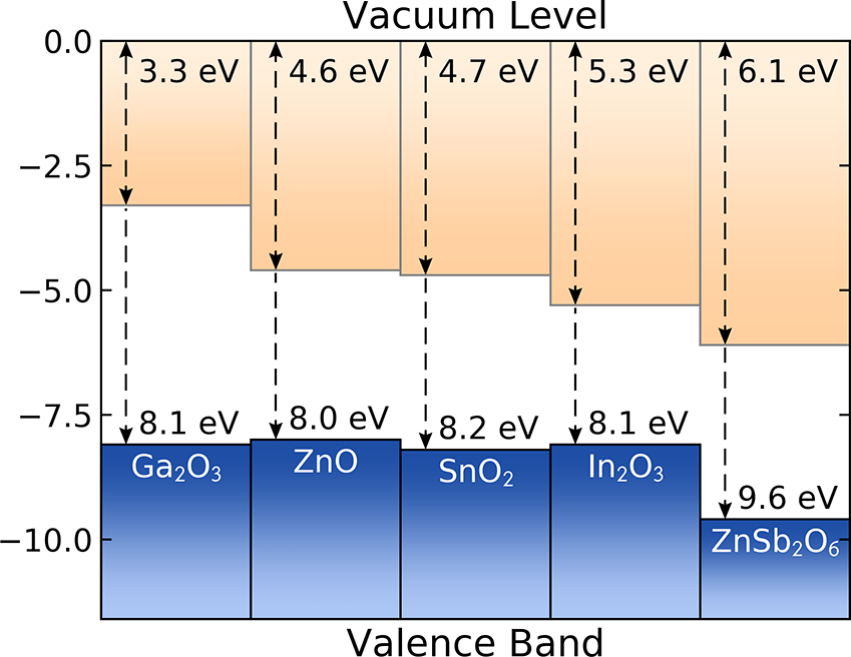
Calculated band alignment of ZnSb_2_O_6_ compared against common TCOs.^37,44,45^

Having verified our computational predictions through single-crystal growth and characterization, we investigated the potential of ZnSb_2_O_6_ as a transparent electrode by calculating its band alignment and comparing to those of existing TCOs, shown in Figure 6. Our calculations reveal an ionization potential (IP) and electron affinity (EA) of 9.6 and 6.1 eV, respectively. Sb 5*s* states contribute strongly to the CBM, much like *ns* states in the other post-transition metal TCOs, but sit lower in energy due to the increased distance from the nucleus and improved shielding of effective charge by core electrons. Therefore, the EA of ZnSb_2_O_6_ is significantly greater than that of the industry-leading TCOs. Upon Ga-doping, the Fermi level is predicted to sit above the CBM, which means a work function nearly 1 eV larger than that of In_2_O_3_ could be achieved. This large work function has tremendous implications in organic photovoltaics (OPVs), which rely entirely on the charge extraction capability of the positive and negative electrodes. A transparent anode with a large electron affinity allows for closer band alignment to low-lying HOMOs (highest occupied molecular orbitals) in OPV devices, providing better Ohmic contacts, an increase in output voltage, and an enhanced device efficiency.^46,47^ Furthermore, replacing organic hole-extracting layers like PEDOT:PSS with a metal oxide like ZnSb_2_O_6_ could help to reduce the corrosion on the electrode.^48^ To engineer large work functions in existing TCOs, modulation of the conduction band is required by alloying with heavy, and sometimes toxic, elements (for example, In_2-*x*_Tl_*x*_O_3_ and Sn_1-*x*_Pb_*x*_O_2_).^37,49^ Exploiting the native band alignment in ZnSb_2_O_6_ is a much cheaper, safer, and easier way of incorporating a large work function material into devices.

We have used ab initio calculations to confirm that ZnSb_2_O_6_ is an earth-abundant transparent conducting oxide, which we have successfully grown in single-crystal form via chemical vapor transport. By studying the intrinsic and extrinsic defect chemistry, we were able to identify an effective doping strategy in order to realize degenerate conductivity through Ga-doping. We have used state-of-the-art packages to predict carrier concentrations and to calculate electron scattering rates, giving a more accurate prediction of charge transport properties that goes beyond the constant relaxation time approximation. Overall, we find good qualitative agreement between these predictions and our single crystals, with our best samples achieving carrier concentrations in excess of 2 × 10^20^ cm^–3^, electron mobility up to 49 cm^2^ V^–1^ s^–1^, and conductivity of 1890 S cm^–1^. The optical behavior of ZnSb_2_O_6_ is also promising, with a direct (forbidden) band gap of around 3.4 eV and a wider predicted optical gap up to 4.2 eV, although the slight blue coloration upon Ga-doping requires further investigation. The next logical step is to develop a thin-film deposition process for Ga-doped ZnSb_2_O_6_, in order to test its performance when incorporated into a device, and to better understand the relationship between charge carrier concentration, transport properties, and optical band gap. Overall, this discovery is a significant milestone in the development of earth-abundant transparent conductors, offering a high-performance alternative to industry standard materials with a unique band edge energy alignment, and opens the door to a whole family of Sb(V)-based transparent conducting oxides.

## Notes

Further experimental and computational data can be found at DOI: 10.5281/zenodo.7034578.

## Supporting Information Available

THz spectroscopy, HAXPES survey and core levels, EDS spectra, an extended discussion intrinsic defects, lattice parameters, hQSGW band structure, calculated material properties used for charge transport calculations, directional dependence plots of charge carrier mobility, and Al and F transition level diagrams, including eq S1, Tables S1–S3, and Figures S1–S8 (PDF)

## References

[ref1] Granqvist C.; Hultåker A. Transparent and conducting ITO films: new developments and applications. Thin Solid Films 2002, 411, 1–5. 10.1016/S0040-6090(02)00163-3.

[ref2] Swallow J. E. N.; Williamson B. A. D.; Whittles T. J.; Birkett M.; Featherstone T. J.; Peng N.; Abbott A.; Farnworth M.; Cheetham K. J.; Warren P.; Scanlon D. O.; Dhanak V. R.; Veal T. D. Self-Compensation in Transparent Conducting F-Doped SnO_2_. Adv. Funct. Mater. 2018, 28, 1701900 10.1002/adfm.201701900.

[ref3] Li J.; Sathasivam S.; Taylor A.; Carmalt C. J.; Parkin I. P. Single step route to highly transparent, conductive and hazy aluminium doped zinc oxide films. RSC Adv. 2018, 8, 42300–42307. 10.1039/C8RA09338E. 35558400 PMC9092156

[ref4] Bhachu D. S.; Scanlon D. O.; Sankar G.; Veal T. D.; Egdell R. G.; Cibin G.; Dent A. J.; Knapp C. E.; Carmalt C. J.; Parkin I. P. Origin of High Mobility in Molybdenum-Doped Indium Oxide. Chem. Mater. 2015, 27, 2788–2796. 10.1021/cm503896h.

[ref5] Swallow J. E. N.; et al. Resonant doping for high mobility transparent conductors: the case of Mo-doped In_2_O_3_. Materials Horizons 2020, 7, 236–243. 10.1039/C9MH01014A.

[ref6] Koida T.; Ueno Y.; Shibata H. In_2_O_3_-Based Transparent Conducting Oxide Films with High Electron Mobility Fabricated at Low Process Temperatures. Physica Status Solidi (A) 2018, 215, 1700506 10.1002/pssa.201700506.

[ref7] Dixon S. C.; Sathasivam S.; Williamson B. A. D.; Scanlon D. O.; Carmalt C. J.; Parkin I. P. Transparent conducting n-type ZnO:Sc – synthesis, optoelectronic properties and theoretical insight. J. Mater. Chem. C 2017, 5, 7585–7597. 10.1039/C7TC02389H.

[ref8] Williamson B. A. D.; et al. Resonant Ta Doping for Enhanced Mobility in Transparent Conducting SnO_2_. Chem. Mater. 2020, 32, 1964–1973. 10.1021/acs.chemmater.9b04845. 32296264 PMC7147269

[ref9] Zhang L.; Zhou Y.; Guo L.; Zhao W.; Barnes A.; Zhang H.-T.; Eaton C.; Zheng Y.; Brahlek M.; Haneef H. F.; Podraza N. J.; Chan M. H. W.; Gopalan V.; Rabe K. M.; Engel-Herbert R. Correlated metals as transparent conductors. Nat. Mater. 2016, 15, 204–210. 10.1038/nmat4493. 26657329

[ref10] Kim H. J.; Kim U.; Kim H. M.; Kim T. H.; Mun H. S.; Jeon B.-G.; Hong K. T.; Lee W.-J.; Ju C.; Kim K. H.; Char K. High Mobility in a Stable Transparent Perovskite Oxide. Appl. Phys. Express 2012, 5, 061102 10.1143/APEX.5.061102.

[ref11] Janowitz C.; Scherer V.; Mohamed M.; Krapf A.; Dwelk H.; Manzke R.; Galazka Z.; Uecker R.; Irmscher K.; Fornari R.; Michling M.; Schmeißer D.; Weber J. R.; Varley J. B.; Van de Walle C. G. Experimental electronic structure of In_2_O_3_and Ga_2_O_3_. New J. Phys. 2011, 13, 085014 10.1088/1367-2630/13/8/085014.

[ref12] Shannon R.; Gillson J.; Bouchard R. Single crystal synthesis and electrical properties of CdSnO_3_, Cd_2_SnO_4_, In_2_TeO_6_ and CdIn_2_O_4_. J. Phys. Chem. Solids 1977, 38, 877–881. 10.1016/0022-3697(77)90126-3.

[ref13] Mizoguchi H.; Woodward P. M. Electronic Structure Studies of Main Group Oxides Possessing Edge-Sharing Octahedra: Implications for the Design of Transparent Conducting Oxides. Chem. Mater. 2004, 16, 5233–5248. 10.1021/cm049249w.

[ref14] Hautier G.; Miglio A.; Waroquiers D.; Rignanese G.-M.; Gonze X. How Does Chemistry Influence Electron Effective Mass in Oxides? A High-Throughput Computational Analysis. Chem. Mater. 2014, 26, 5447–5458. 10.1021/cm404079a.

[ref15] Kikuchi N.; Hosono H.; Kawazoe H.; Tanegashima O.; Ota I.; Kimura Y. Carrier Generation in Wide-Gap Conductor, Zinc Antimonate. J. Am. Ceram. Soc. 2005, 88, 2793–2797. 10.1111/j.1551-2916.2005.00528.x.

[ref16] Tamaki J.; Yamada Y.; Yamamoto Y.; Matsuoka M.; Ota I. Sensing properties to dilute hydrogen sulfide of ZnSb_2_O_6_ thick-film prepared by dip-coating method. Sens. Actuators, B 2000, 66, 70–73. 10.1016/S0925-4005(99)00408-6.

[ref17] Zhu B.; Xie C.; Wang A.; Zeng D.; Hu M.; Wang W. Electrical conductivity and gas sensitivity of Zn–Sb–O thick films. Mater. Res. Bull. 2004, 39, 409–415. 10.1016/j.materresbull.2003.10.011.

[ref18] Potter D. Zinc-based thin films for transparent conducting oxide applications. Ph.D. thesis, University College London, 2018.

[ref19] Li J.; Du K.; Lai Y.; Chen Y.; Zhang Z. ZnSb_2_O_6_: an advanced anode material for Li-ion batteries. J. Mater. Chem. A 2017, 5, 10843–10848. 10.1039/C7TA02290E.

[ref20] U.S. Geological Survey: Mineral commodity summaries 2022; U.S. Geological Survey 2022; p 202.

[ref21] Zhang G.-X.; Reilly A. M.; Tkatchenko A.; Scheffler M. Performance of various density-functional approximations for cohesive properties of 64 bulk solids. New J. Phys. 2018, 20, 063020 10.1088/1367-2630/aac7f0.

[ref22] Momma K.; Izumi F. *VESTA*: a three-dimensional visualization system for electronic and structural analysis. J. Appl. Crystallogr. 2008, 41, 653–658. 10.1107/S0021889808012016.

[ref23] Shannon R. D. Revised effective ionic radii and systematic studies of interatomic distances in halides and chalcogenides. Acta Crystallogr., Sect. A 1976, 32, 751–767. 10.1107/S0567739476001551.

[ref24] Toby B. H.; Von Dreele R. B. GSAS-II: the genesis of a modern open-source all purpose crystallography software package. J. Appl. Crystallogr. 2013, 46, 544–549. 10.1107/S0021889813003531.

[ref25] Lebens-Higgins Z.; Scanlon D.; Paik H.; Sallis S.; Nie Y.; Uchida M.; Quackenbush N.; Wahila M.; Sterbinsky G.; Arena D. A.; Woicik J.; Schlom D.; Piper L. Direct Observation of Electrostatically Driven Band Gap Renormalization in a Degenerate Perovskite Transparent Conducting Oxide. Phys. Rev. Lett. 2016, 116, 027602 10.1103/PhysRevLett.116.027602. 26824566

[ref26] Walsh A.; Da Silva J. L. F.; Wei S.-H.; Korber C.; Klein A.; Piper L. F. J.; DeMasi A.; Smith K. E.; Panaccione G.; Torelli P.; Payne D. J.; Bourlange A.; Egdell R. G. Nature of the Band Gap of In_2_O_3_ Revealed by First-Principles Calculations and X-Ray Spectroscopy. Phys. Rev. Lett. 2008, 100, 167402 10.1103/PhysRevLett.100.167402. 18518246

[ref27] Jackson A. J.; Ganose A. M.; Regoutz A.; Egdell R. G.; Scanlon D. O. Galore: Broadening and weighting for simulation of photoelectron spectroscopy. J. Open Source Software 2018, 3, 773 10.21105/joss.00773.

[ref28] Yeh J.; Lindau I. Atomic subshell photoionization cross sections and asymmetry parameters: Z1–103. Atomic Data and Nuclear Data Tables 1985, 32, 1–155. 10.1016/0092-640X(85)90016-6.

[ref29] Oba F.; Togo A.; Tanaka I.; Paier J.; Kresse G. Defect energetics in ZnO: A hybrid Hartree-Fock density functional study. Phys. Rev. B 2008, 77, 245202 10.1103/PhysRevB.77.245202.

[ref30] Vasheghani Farahani S. K.; Veal T. D.; Mudd J. J.; Scanlon D. O.; Watson G. W.; Bierwagen O.; White M. E.; Speck J. S.; McConville C. F. Valence-band density of states and surface electron accumulation in epitaxial SnO_2_ films. Phys. Rev. B 2014, 90, 155413 10.1103/PhysRevB.90.155413.

[ref31] Buckeridge J.; Scanlon D.; Walsh A.; Catlow C. Automated procedure to determine the thermodynamic stability of a material and the range of chemical potentials necessary for its formation relative to competing phases and compounds. Comput. Phys. Commun. 2014, 185, 330–338. 10.1016/j.cpc.2013.08.026.

[ref32] Mott N. F. Metal-Insulator Transition. Rev. Mod. Phys. 1968, 40, 677–683. 10.1103/RevModPhys.40.677.

[ref33] Edwards P. P.; Sienko M. J. Universality aspects of the metal-nonmetal transition in condensed media. Phys. Rev. B 1978, 17, 2575 10.1103/PhysRevB.17.2575.

[ref34] Scanlon D. O.; Kehoe A. B.; Watson G. W.; Jones M. O.; David W. I. F.; Payne D. J.; Egdell R. G.; Edwards P. P.; Walsh A. Nature of the Band Gap and Origin of the Conductivity of PbO_2_ Revealed by Theory and Experiment. Phys. Rev. Lett. 2011, 107, 246402 10.1103/PhysRevLett.107.246402. 22243014

[ref35] Spooner K. B.; Ganose A. M.; Scanlon D. O. Assessing the limitations of transparent conducting oxides as thermoelectrics. J. Mater. Chem. A 2020, 8, 11948–11957. 10.1039/D0TA02247K.

[ref36] Chatratin I.; Sabino F. P.; Reunchan P.; Limpijumnong S.; Varley J. B.; Van de Walle C. G.; Janotti A. Role of point defects in the electrical and optical properties of In_2_O_3_. Phys. Rev. Mater. 2019, 3, 074604 10.1103/PhysRevMaterials.3.074604.

[ref37] Scanlon D. O.; Watson G. W. On the possibility of p-type SnO_2_. J. Mater. Chem. 2012, 22, 25236 10.1039/c2jm34352e.

[ref38] Janotti A.; Van de Walle C. G. Native point defects in ZnO. Phys. Rev. B 2007, 76, 165202 10.1103/PhysRevB.76.165202.

[ref39] Nguyen V. H.; Gottlieb U.; Valla A.; Muñoz D.; Bellet D.; Muñoz-Rojas D. Electron tunneling through grain boundaries in transparent conductive oxides and implications for electrical conductivity: the case of ZnO:Al thin films. Materials Horizons 2018, 5, 715–726. 10.1039/C8MH00402A.

[ref40] Ganose A. M.; Park J.; Faghaninia A.; Woods-Robinson R.; Persson K. A.; Jain A. Efficient calculation of carrier scattering rates from first principles. Nat. Commun. 2021, 12, 2222 10.1038/s41467-021-22440-5. 33850113 PMC8044096

[ref41] Ellmer K. Past achievements and future challenges in the development of optically transparent electrodes. Nat. Photonics 2012, 6, 809–817. 10.1038/nphoton.2012.282.

[ref42] Murgatroyd P. A. E.; Smiles M. J.; Savory C. N.; Shalvey T. P.; Swallow J. E. N.; Fleck N.; Robertson C. M.; Jäckel F.; Alaria J.; Major J. D.; Scanlon D. O.; Veal T. D. GeSe: Optical Spectroscopy and Theoretical Study of a van der Waals Solar Absorber. Chem. Mater. 2020, 32, 3245–3253. 10.1021/acs.chemmater.0c00453. 32308255 PMC7161679

[ref43] Ha V.-A.; Waroquiers D.; Rignanese G.-M.; Hautier G. Influence of the second gap on the transparency of transparent conducting oxides: An ab initio study. Appl. Phys. Lett. 2016, 108, 201902 10.1063/1.4950803.

[ref44] Zhang J.; Willis J.; Yang Z.; Lian X.; Chen W.; Wang L.-S.; Xu X.; Lee T.-L.; Chen L.; Scanlon D. O.; Zhang K. H. Deep UV transparent conductive oxide thin films realized through degenerately doped wide-bandgap gallium oxide. Cell Rep. Phys. Sci. 2022, 3, 100801 10.1016/j.xcrp.2022.100801.

[ref45] Höffling B.; Schleife A.; Rödl C.; Bechstedt F. Band discontinuities at Si-TCO interfaces from quasiparticle calculations: Comparison of two alignment approaches. Phys. Rev. B 2012, 85, 035305 10.1103/PhysRevB.85.035305.

[ref46] Greiner M. T.; Lu Z.-H. Thin-film metal oxides in organic semiconductor devices: their electronic structures, work functions and interfaces. NPG Asia Materials 2013, 5, e55–e55a. 10.1038/am.2013.29.

[ref47] Cao W.; Xue J. Recent progress in organic photovoltaics: device architecture and optical design. Energy Environ. Sci. 2014, 7, 2123 10.1039/c4ee00260a.

[ref48] Kim S.; Saeed M. A.; Kim S. H.; Shim J. W. Enhanced hole selecting behavior of WO3 interlayers for efficient indoor organic photovoltaics with high fill-factor. Appl. Surf. Sci. 2020, 527, 146840 10.1016/j.apsusc.2020.146840.

[ref49] Ganose A. M.; Scanlon D. O. Band gap and work function tailoring of SnO_2_ for improved transparent conducting ability in photovoltaics. J. Mater. Chem. C 2016, 4, 1467–1475. 10.1039/C5TC04089B.

