# Supplementary material for: Computational Prediction and Experimental Realization of Earth-Abundant Transparent Conducting Oxide Ga-Doped ZnSb2O6
Source: ACS Energy Lett. 2022 Oct 10;7(11):3807–16. doi: 10.1021/acsenergylett.2c01961 (PMC9664443; doi:10.1021/acsenergylett.2c01961)
Supplement: Supplementary file 1 — nz2c01961_si_001.pdf [file nz2c01961_si_001.pdf]

SI: Computational Prediction and Experimental Realisation of Earth Abundant Transparent Conducting Oxide Ga-doped $ZnSb_2O_6$

Adam J. Jackson[**,†] Benjamin J. Parrett[**,‡,¶] Joe Willis[**,¶,§,‖] Alex M. Ganose,[⊥] W. W. Winnie Leung,[§] Yuhan Liu,[‡,§] Benjamin A. D. Williamson,[#] Timur K. Kim,[¶] Moritz Hoesch,[‡,¶] Larissa S. I. Veiga,[‡,¶] Raman Kalra,[§] Jens Neu,[@] Charles A. Schmuttenmaer[***,@] Tien-Lin Lee,[¶] Anna Regoutz,[§,¶] Tung-Chun Lee,[§,△] Tim D. Veal,[∇] Robert G. Palgrave,[§] Robin Perry,[*,‡,††] and David O. Scanlon[*,§,‖]

†*Scientific Computing Dept, Science and Technology Facilities Council, Rutherford Appleton Laboratory, Harwell Science and Innovation Campus, Didcot, Oxfordshire, OX11 0QX, UK.*

‡*London Centre for Nanotechnology and Dept of Physics and Astronomy, University College London, Gordon St, London, WC1E 6BT, UK.*

¶*Diamond Light Source Ltd., Harwell Science and Innovation Campus, Didcot, Oxfordshire, OX11 0DE, UK.*

§*Dept of Chemistry, University College London, 20 Gordon St, London, WC1H 0AJ, UK.*

‖*Thomas Young Centre, University College London, Gower St, London, WC1E 6BT, UK.*

⊥*Dept of Materials, Imperial College London, Exhibition Rd, London, SW7 2AZ, UK.*

#*Dept of Materials Science and Engineering, Norwegian University of Science and Technology (NTNU), Trondheim, 7491, Norway.*

@*Dept of Chemistry, Yale University, New Haven, Connecticut, 06520-8107, USA.*

△*Institute of Materials Discovery, University College London, Malet Place, WC1E 7JE, UK.*

∇*Dept of Physics and Stephenson Institute for Renewable Energy, University of Liverpool, Liverpool, L69 7ZF, UK.*

Supplementary Information

THz spectroscopy

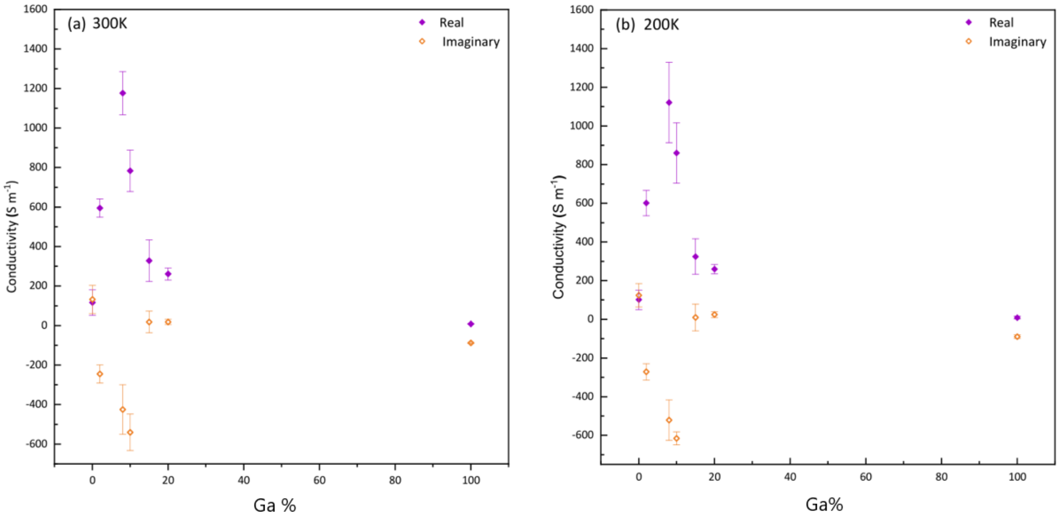

Figure S1: TDS spectra of $Zn_{1\text{-}x}Ga_xSb_2O_6$ solid solutions at 300 K and 200 K.

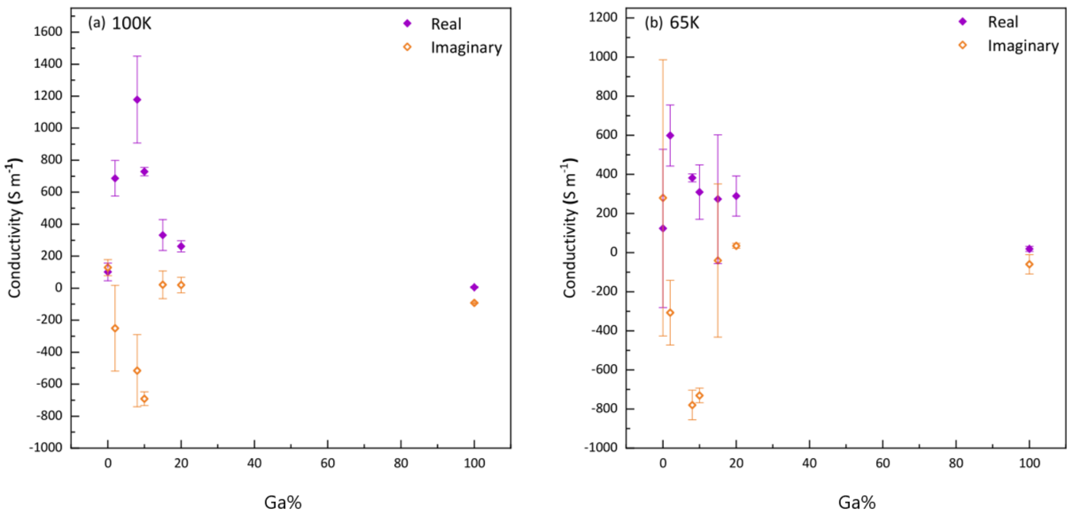

Figure S2: TDS spectra of $Zn_{1\text{-}x}Ga_xSb_2O_6$ solid solutions at 100 K and 65 K. Error bars become significantly larger at lower temperatures.

As synthesised $Zn_{1\text{-}x}Ga_xSb_2O_6$ solid solutions with nominal compositions of 2%, 8%, 10%, 15% and 20% Ga were measured using THz-domain spectroscopy (TDS) in order to obtain electrical conductivities. The spectrometer used a titanium sapphire laser as the source of

ultrafast optical pulses (50 fs) with a wavelength of 800 nm. The generated pulses had a bandwidth of 0.3 THz to 3 THz, and the data was processed at 1 THz. Figures S1 and S2 show the electrical conductivity of these solid solutions at a range of temperatures, which are supportive of metallic-like conductivity upon Ga incorporation into the tri-rutile $ZnSb_2O_6$ structure. The data at 200 K and 300 K are also overlaid on the conductivity plot in the main text.

Conductivity of the pure $ZnSb_2O_6$ sample is low at all temperatures, supportive of the defect chemistry that there are no shallow, intrinsic donors able to provide intrinsic, degenerate conductivity. As the nominal percentage of Ga increases, conductivity rises to a maximum around 1200 $\mathrm{S\,cm^{-1}}$, after which it begins to tail off. This is about 60% of the conductivity achieved in the most heavily doped single crystal. Considering the grain boundaries and extended defects present in powder samples, this is as expected.

HAXPES

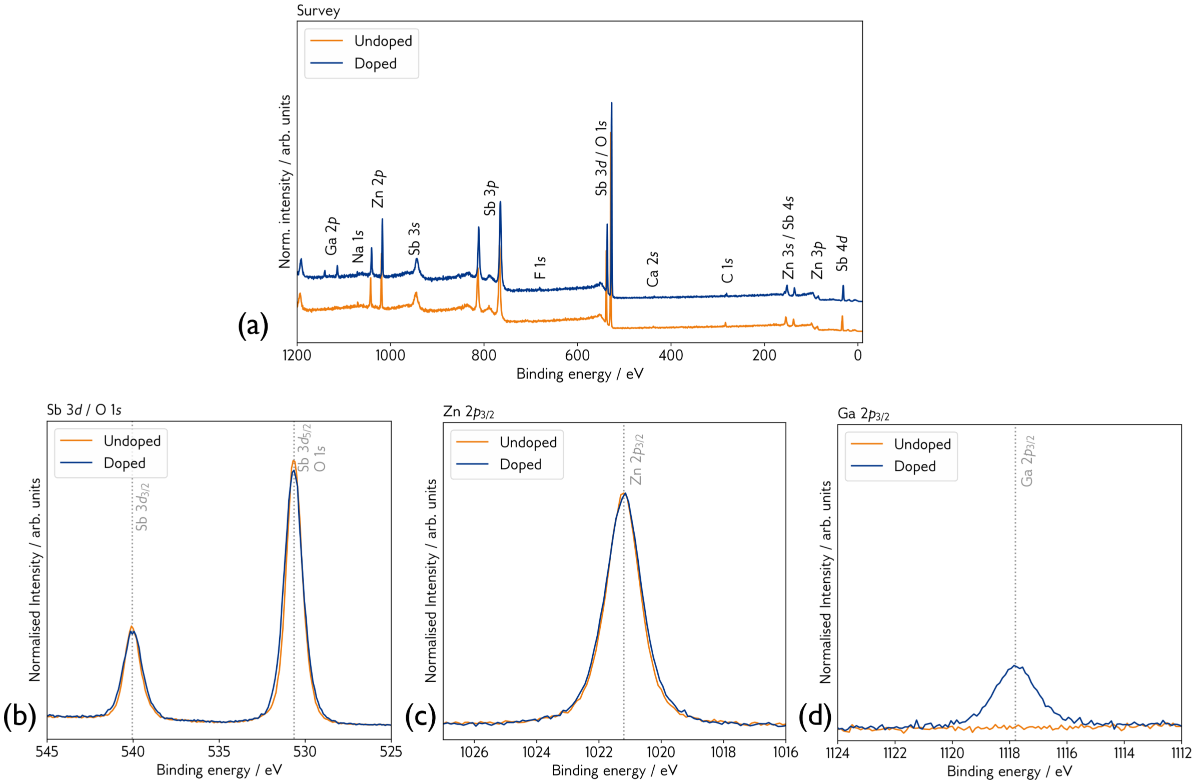

Figure S3: (a) Survey spectra of $ZnSb_2O_6$ single crystals, showing all expected core levels, as well as very minor Ca and F contamination in the crystals; (b) Sb 3*d* and O 1*s* core level spectra of $ZnSb_2O_6$ single crystals; (c) Zn 2*p* core level spectra of $ZnSb_2O_6$ single crystals; (d) Ga 2*p* core level spectra of $ZnSb_2O_6$ single crystals, clearly demonstrating Ga is not present in the nominally undoped crystal and present in the doped crystal.

Figure S3 shows the HAXPES spectra for the undoped and doped single crystals. The survey spectra show the appearance of all expected Zn, Sb and O core levels, as well as minor contaminants. The Zn 2*p*, Sb 3*d* and O 1*s* core spectra are very similar in both samples (we note that the Sb $3d_{5/2}$ and O 1*s* levels overlap at a binding energy of around 531 eV), while there is clear evidence of the presence of Ga at a binding energy of around 1118 eV in Figure S3d in the doped sample, and the absence of Ga in the undoped sample. The fact that we also observe conduction band emission in the doped sample and not the undoped sample suggests strongly that it is the Ga that is contributing electrons and raising the Fermi level.

EDS

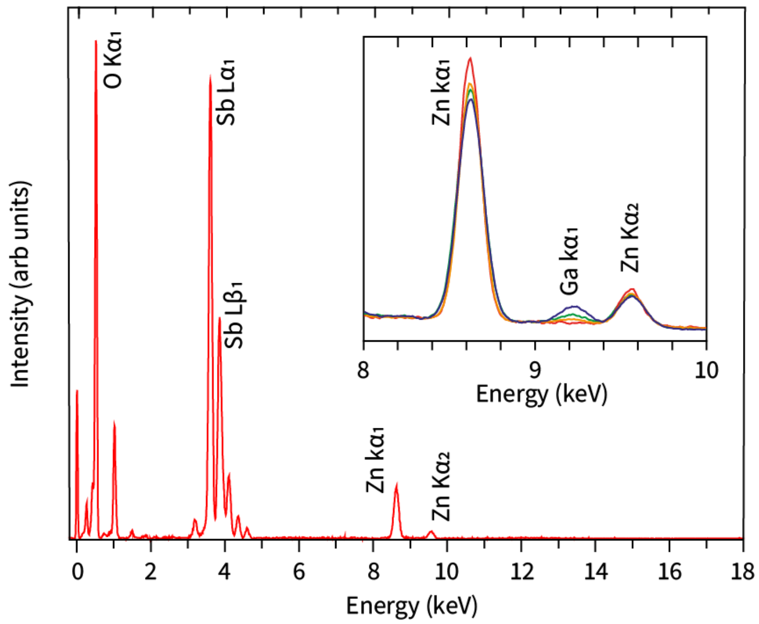

Figure S4: EDS spectrum for nominally undoped $ZnSb_2O_6$ (red). Inset displays expanded energy range 8 keV to 10 keV with doped crystals overlaid showing the change in intesity between Ga Kα_1 and Zn Kα_1. Background subtracted and data normalised to Sb Lα intesity of each crystal. Moving from orange to green to blue denotes heavier Ga-doping.

Extended defect discussion

Cation vacancies

The zinc vacancy in $ZnSb_2O_6$ has formation energy of 6.58 eV in the neutral charge state, with transition levels from the (0/1-) and (1-/2-) charge states occurring at Fermi levels of 1.20 eV and 1.63 eV, respectively. This is a deep acceptor defect that displays polaronic behaviour, as shown in the charge density plot in Figure S5. In the neutral charge state, two holes are stabilised, which reduces to one upon the acceptance of an electron from the VBM, and to none upon the acceptance of a second electron. This is analogous to the behaviour seen in the zinc vacancy in ZnO.[1]

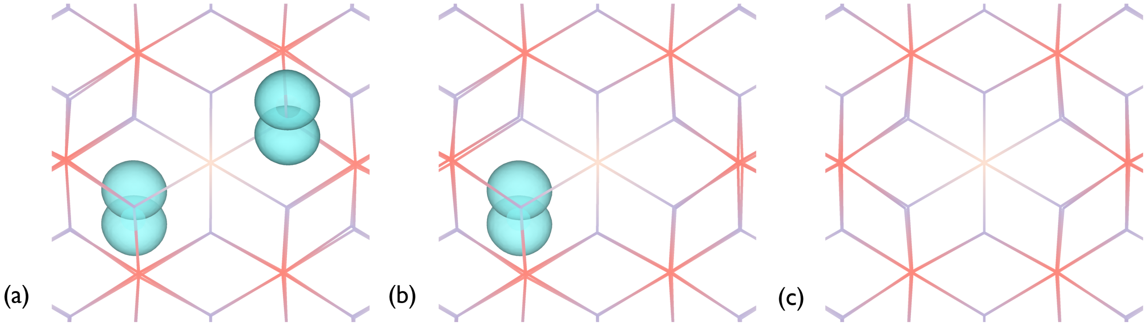

Figure S5: Zinc vacancy and corresponding hole density in various charge states. (a) neutral; (b) 1-; (c) 2-. Isosurface plotted with a density of 0.07 electron $Å^{-3}$.

The antimony vacancy has much higher formation energies (over 13 eV in the neutral charge state), and is not plotted on the transition level diagram in the main text. It too is a deep acceptor defect that displays polaronic behaviour, stabilising five holes around the vacancy, which are in turn reduced as the defect accepts more electrons from the VBM.

Oxygen vacancies

There are two distinct oxygen environments in $ZnSb_2O_6$, shown in Figure 1b. In the first (light blue O atom), there are two equal Sb-O bonds of length 1.99 Å and a single Zn-O bond of 2.07 Å. In the second, (dark blue O atom), the Sb-O bonds are of different lengths,

1.97 Å for the Sb in the edge-sharing polyhedron and 1.98 Å for the Sb in the corner-sharing polyhedron, while the Zn-O bond is longer at 2.09 Å. The formation energies of the neutral vacancy are 1.92 eV and 1.98 eV, respectively, with both stabilising the +1 charge state for small Fermi level range (0.03 eV and 0.05 eV respectively).

In environment one, upon V_O generation, the two Sb atoms relax inwards by 2.6%, while the Zn relaxes outwards by 6.8%. Upon ionisation to V_O^+, the two Sb atoms relax outwards by 7.4% from their position in the neutral vacancy, and the Zn relaxes outwards by a further 0.7%. Ionising again to V_O^{++} causes a further 6.8% outwards relaxation of the Sb atoms and another 0.8% for the Zn atom.

In environment two, when the neutral vacancy forms, the Sb atom with the shorter bond length relaxes inwards by 10.9%, while the other Sb atom relaxes outwards by 3.7% and the Zn atom relaxes outwards by 8%. The Zn atom remains in approximately the same position regardless of charge state. Upon ionisation to V_O^+, the first Sb atom relaxes outwards again by 11.5%, surpassing its original position, while the other Sb atom relaxes outwards by a further 8.0%. Then after the second ionisation, the first Sb relaxes away by 13.5% and the second by a further 2.7%.

In both cases, Sb is preferentially attracted towards the neutral vacancy over Zn, presumably due to the higher charge on the cation and the smaller ionic radius. The relatively equally-sized steps in inward and outward relaxation as a function of charge state in both cases stabilises the narrow formation window of V_O^+ in $ZnSb_2O_6$, similar to what is observed in In_2O_3.[2] This is different to the negative-U behaviour observed in SnO_2 and ZnO,[3,4] which is driven by the much larger and non-stepwise change in bond lengths as a function of charge state.

Miscellaneous

Table S1: Lattice parameters of $ZnSb_2O_6$ resolved from experiment and simulations. Asterisks denote results from this work.

Method	a / Å	c / Å	a/c
PXRD*	4.6793	9.2906	0.5037
PBE0*	4.6740	9.2585	0.5048
PBEsol*	4.6921	9.3388	0.5024
Bystroem et al	4.67	9.26	0.50
Nishiyama et al[5]	4.668	9.265	0.504
Kikuchi et al[6]	4.68	9.29	0.50

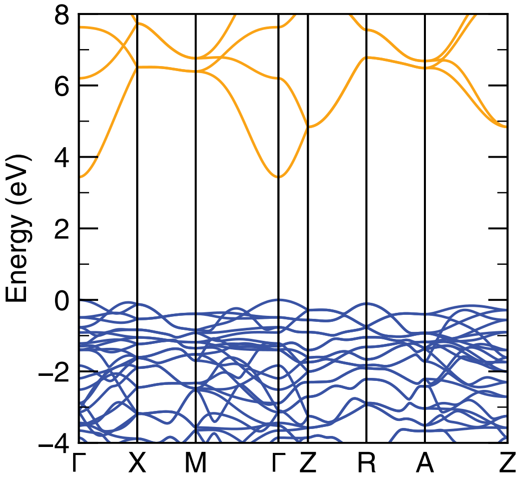

Figure S6: Band structure computed with quasi-particle self-consistent GW using 80% scaling of self-energy (hQSGW).

All AMSET calculations were performed with the default settings, with the addition of free-carrier screening in the polar optical phonon matrix element (`free_carrier_screening: true`).

$$\text{High-frequency dielectric constant } (\epsilon_0) = \begin{bmatrix} 3.15 & 0 & 0 \\ 0 & 3.15 & 0 \\ 0 & 0 & 3.45 \end{bmatrix}$$

$$\text{Static dielectric constant } (\epsilon_0) = \begin{bmatrix} 12.16 & 0 & 0 \\ 0 & 12.16 & 0 \\ 0 & 0 & 8.86 \end{bmatrix}$$

$$\text{Elastic constant (GPa)} = \begin{bmatrix} 234 & 159 & 138 & 0 & 0 & 0 \\ 159 & 234 & 138 & 0 & 0 & 0 \\ 138 & 138 & 393 & 0 & 0 & 0 \\ 0 & 0 & 0 & 178 & 0 & 0 \\ 0 & 0 & 0 & 0 & 86 & 0 \\ 0 & 0 & 0 & 0 & 0 & 86 \end{bmatrix}$$

$$\text{Polar optical phonon frequency (THz)} = 10.767$$

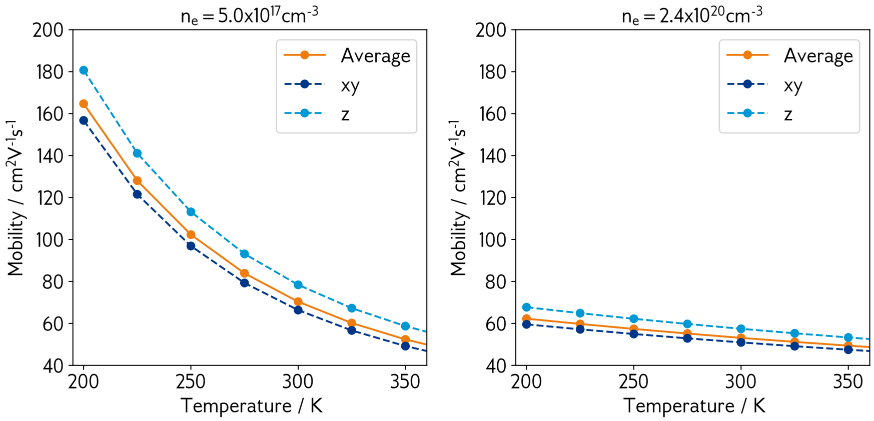

Figure S7: Directional mobility of $ZnSb_2O_6$ at room temperature at two different carrier concentrations.

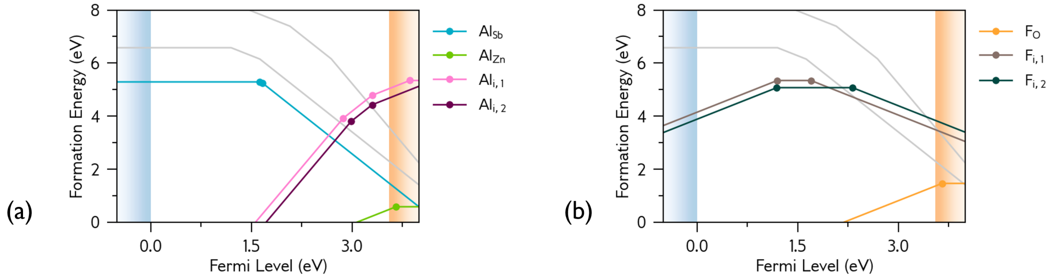

Figure S8: (a) Al-doping and (b) F-doping of $ZnSb_2O_6$. Grey faded lines show the native p-type acceptors V_{Zn} and Zn_{Sb}.

Methodology

Computational Methods

Geometry Optimisation and Electronic Structure

Density functional theory calculations were primarily performed within the plane-wave periodic code VASP,[7–10] which uses the projector augmented wave method to describe the interactions between valence and core states,[11,12] details of which can be found in Table S2. A plane-wave energy cut-off of 500 eV was used for all calculations, and a $7 \times 7 \times 4$ Γ-centred $\mathbf{k}$-point mesh was employed. The PBE0[13,14] hybrid exchange correlation functional was used because it has been shown to accurately reproduce the band gap of rutile-structured SnO_2.[3,15,16] $ZnSb_2O_6$ shares a rutile-based crystal structure, so we expected PBE0 to perform well in this case – the calculated fundamental gap is within 0.15 eV of the experimentally derived value, closing to within 0.03 eV when used within the hQSGW approach. The static dielectric constant was calculated with density functional perturbation theory (DFPT) using the PBEsol functional.[17–19] Effective masses and band structures were calculated and plotted using the SUMO code.[20]

Table S2: Explicitly treated valence electrons in DFT calculations.

O	F	Al	Zn	Ga	Sb
$2s^2 2p^4$	$2s^2 2p^5$	$3s^2 3p^1$	$3d^{10} 4s^2$	$4s^2 4p^1$	$5s^2 5p^3$

Additionally, a quasiparticle calculation was performed using the "hybrid quasiparticle self-consistent GW" (hQSGW) method as implemented in the QUESTAAL code using a linearised muffin-tin orbital (LMTO) basis set. In this hybrid approach a converged self-energy is obtained by the quasiparticle self-consistent GW (QSGW) approach, and an empirical 80% of the self-energy is combined with the LDA self-energy to obtain a prediction of the semiconductor bandgap.[21–23] This correction is routinely used to account for neglected interactions and improve the accuracy of bandgap estimations.[24,25] The lattice parameters and

atomic positions were used from the results of the PBE0 geometry optimisation. The LMTO basis set was generated with the recommended parameters in QUESTAAL, using a $10\,\mathrm{Ry}^{1/2}$ cut-off for the interstitial mesh in the one-particle Hamiltonian steps, $2.7\,\mathrm{Ry}^{1/2}$ cut-off for the interstitial mesh of the two-particle objects and $3.3\,\mathrm{Ry}^{1/2}$ cut-off for the basis envelope functions in GW steps. The $7 \times 7 \times 4$ **k**-point grid used for the DFT calculations was reduced to $3 \times 3 \times 2$ for the GW steps. Effective masses were determined by quadratic fitting to band structure data, using tools in the QUESTAAL package.

Optical calculations

The frequency-dependent dielectric matrix was calculated in VASP using the PBE0 functional, a well-converged **k**-point grid of $7 \times 7 \times 4$, and a low smearing value. The frequency dependent matrix was converted into an absorption spectrum using sumo,[20] to which a small amount of Gaussian smearing was added to improve visualisation.

Defect calculations

Stable competing phases within the Zn-Sb-O chemical potential space were relaxed using the PBE0 functional, a plane-wave energy cut-off of 500 eV and a converged **k**-point mesh until the forces were reduced to below $1 \times 10^{-2}\,\mathrm{eV\,Å}^{-1}$. The ground state energies were used to identify the chemical potential limits that bound the thermodynamic stability of $ZnSb_2O_6$, using the program CPLAP.[26] The chemical potential of oxygen was set as the dependent variable in the CPLAP analysis, as this can be most readily changed in experiment through the use of partial pressures. The chemical potential limits from the thermodynamic stability calculations can be found in Table S3.

A $2 \times 2 \times 1$ supercell (72 atoms) of the primitive cell was generated as an approximately cubic template for defect calculations (9.35 Å by 9.35 Å by 9.26 Å). There are two distinct cation sites in the $P4_2/mnm$ space group (Figure 1b), one occupied by Zn (pale orange) and the other by Sb (dark orange) in $ZnSb_2O_6$, and two distinct anion sites (pale and dark

Table S3: Chemical potential limits (in eV) of each element considered in this defect study. Asterisk denotes the limits used in the transition level diagrams in the main text.

	Sb	Zn	O	Al	Ga	F
A	-0.4661	-1.206	-1.9432	-5.1465	-2.2096	-3.1588
B*	-0.669	-1.6117	-1.808	-5.3493	-2.4124	-2.9559
C	-2.7628	-3.7055	-0.7611	-6.9196	-3.9828	-1.909
D	-4.6655	-4.4666	0	-8.0613	-5.1244	-1.5285
E	-5.3273	-3.143	0	-8.0613	-5.1244	-2.1903

blue), both occupied by O. Two interstitial candidate sites were identified: "i1" on the $8h$ Wyckoff site between two Zn and two Sb atoms, and "i2" on the $4c$ Wyckoff site between two Zn atoms. All supercells were optimised to reduce forces below 1×10^{-2} eV Å^{-1}, keeping the lattice vectors constant while allowing ionic coordinates to move. Supercell calculations were performed using a Γ-centred $2 \times 2 \times 2$ **k**-point mesh.

The Gibbs free energy of formation for each defect D with charge q in a given chemical environment μ and at a given Fermi energy E_F (relative to the VBM) is approximated as:[27]

$$\Delta G_f(D, q, \mu, E_F) = (E_{D,q} - E_{\text{host}}) + \sum_i n_i(E_i^{\text{ref}} + \mu_i) + q(E_F + \epsilon_{\text{VBM,host}}) + E_{\text{sc-corr}}(D, q). \quad (1)$$

To account for changes in composition n of each element i, the DFT total energy of a standard elemental reference E_i^{ref} is combined with a relative value of chemical potential μ_i. Post-processing supercell corrections are applied to counteract the effects of using a finite supercell, and consist of: potential alignment correction, to account for the shift in eigenvalues between a charged and non-charged supercell;[28] anisotropic image charge correction, developed by Murphy and Hine from the Makov-Payne method, a finite-size correction that removes the Coulombic repulsion between periodic images of defective supercells by treating the defect as a periodic point charge in a dielectric medium;[29,30] and a band filling correction, which counteracts the unrealistic filling of the conduction band (emptying of the valence band) in a finite-sized supercell.[28] At a given E_F, the lowest energy charge state

dominates for that defect, and a “transition level” is the point at which two charge states are in thermodynamic equilibrium.

Charge Transport Calculations

Electronic transport properties were calculated using the AMSET package, which solves the linearised Boltzmann transport equation under the relaxation time approximation. Unlike the constant relaxation time approach, AMSET explicitly calculates band and **k**-dependent relaxation times using scattering matrix elements obtained from first principles inputs. Accordingly, AMSET can provide fundamental insights into the strength of scattering processes that limit charge transport, and has demonstrated excellent agreement with experimental measurements of mobility in a range of semiconductors.[31] In the present work, we have included scattering due to polar optical phonons (POP), acoustic deformation potentials (ADP), and ionised impurities (IMP). We have not included piezoelectric scattering as $ZnSb_2O_6$ is centrosymmetric ($P4_2/mnm$ point group symmetry) and therefore does not display piezoelectricity. The primary input for AMSET was a hQSGW band structure calculation on a relatively dense $7 \times 7 \times 4$ **k**-point mesh. To calculate wave function overlaps, we used the wave function coefficients from a PBE0 calculation on the same k-point mesh, performed using VASP. The hQSGW and PBE0 computational methodologies were consistent with the calculations described above. To obtain the transport properties and scattering rates, the electronic band structure and wave function coefficients were interpolated onto a dense $87 \times 87 \times 45$ k-point mesh. One benefit of AMSET compared to state-of-the-art approaches based on density functional perturbation theory combined with Wannier interpolation (DFPT+Wannier) is that scattering rates can be obtained from common materials parameters without requiring an expensive DFPT calculation.[32] The calculated materials parameters (dielectric constants, polar phonon frequency, and elastic constants) along with additional settings used by AMSET are provided in the Supporting Information. The full AMSET methodology, including the scattering matrix elements and interpolation scheme is

given in detail in Ref. 31.

Self Consistent Fermi Level Analysis

To calculate the SCFL of $ZnSb_2O_6$, a `python` based implementation of SC-FERMI was used (which can be found at `https://github.com/bjmorgan/py-sc-fermi`).[33] The required inputs are an electronic density of states of the defect-free system, a temperature, the total cell volume and number of electrons, and the thermodynamic transition levels for all defect species. The SCFL is calculated by recognising that the overall charge of a system must be equal to zero, which must be equal to the concentration of all of the charged defects plus any positive holes and negative electrons. We can construct simultaneous equations using the Fermi-Dirac distribution and the formation energy of a defect (Equation 1), and therefore find the Fermi level that gives overall charge neutrality. A rigorous description of this problem, and how it is implemented in the code, can be found in the original paper.[33]

In our analysis, we calculated the SCFL at the synthesis temperature of $ZnSb_2O_6$ and froze the defect concentrations at this temperature. We then recalculated the SCFL at room temperature, allowing the concentration of the individual charge states of each defect to change, in order to predict the total electron concentration at the temperature at which our experiments were performed.

Band alignment

The core level alignment method was used to calculate the ionisation potential and electron affinity of $ZnSb_2O_6$.[34] A slab-gap model was constructed using the PBE0 relaxed structure and a vacuum and slab thickness of 30 Å using the SURFAXE code.[35] The (110) termination was selected, which has been demonstrated to be the lowest energy surface for rutile SnO_2.[36] The planar average of the electrostatic potential was converged and calculated within SURFAXE, and the plateau of this was taken to be the energy of the vacuum.

Experimental Methods

Solid State Synthesis

Powders of ZnO (Sigma-Aldrich, 4N), Sb_2O_3 (Sigma-Aldrich, 4N) and Ga_2O_3 (Sigma-Aldrich, 5N) were dried and ground together in near stoichiometric molar ratios. The powder was heated for 12 hours at 600 °C in a 400 mbar Ar atmosphere in a sealed quartz tube to pre-react to form the $ZnSb_2O_4$ phase. The 1-2-4 polycrystalline powder was then extracted, reground and heated in an air atmosphere at 800 °C for a further 12 hours for oxidation into the $ZnSb_2O_6$ (1-2-6) phase. This two-step process was necessary to control antimony evaporation during baking,[6] and we typically observed less than 1% Sb loss during the synthesis which could be accurately accounted for by adding excess Sb_2O_3 in the starting materials.

Chemical vapour transport (CVT) was selected for the crystal growth method as it is appropriate for materials with high melting points and low vapour pressures such as ZnO[37] and Ga_2O_3.[38] Advantages include that high purity crystals can be obtained as, due to the closed nature of the growth system, minimal external impurities can be incorporated into the crystals. A sealed quartz ampule was employed using Cl_2 as a transport agent. The Cl_2 was added to the transport ampule via an evacuation rig similar to that described by Binnewies et al.[39] The tube was sealed at a length of 15 cm and placed horizontally at the centre of a two-zone furnace set at 1100 °C and 1000 °C. Since the reaction is endothermic the precursor was placed at the hot end of the ampule. After 200 hr, the powder was fully transported to the cold end in the form of single crystals adhered to the ampule walls. The ampule showed signs of attack during the growth reaction determined by XRD to be cristobalite (SiO_2), a feature not uncommon in CVT reactions using Cl_2 as a transport agent.[40] It is important to note that neither hard X-ray photoelectron spectroscopy (HAXPES) surveys (Figure S3a) nor energy-dispersive X-ray spectroscopy (EDS) measurements (Figure S4) showed any silicon in the crystals. Gallium oxide also had a tendency to react with the ampule walls, and around 20% molar excess was added to the starting powders to compensate.

Characterisation

Samples were cut and polished into cuboid geometries with typical dimensions of 0.8 mm by 0.6 mm by 0.2 mm. The composition of the crystals was determined using an Oxford instruments EDS system built into a JEOL JSM-6060OLV scanning electron microscope (SEM) operating at 20 keV, and the resultant data analysis using the Aztec software from Oxford Instruments (Figure S4). Electrical properties of the crystals were determined using a five-point Hall-bar geometry with Dupont silver-epoxy contacts annealed onto the bars allow Hall effect and resistivity measurements concurrently. Measurements were taken on a Quantum Design PPMS-9 using a 1 mA excitation current in magnetic fields between ± 1 T at temperatures from 350 K to 200 K. The Hall component of the transverse voltage V_{xy} was obtained by extracting the odd function dependence of the transverse resistance with an applied magnetic field. For optical measurements, a Shimadzu Solid UV-Vis-IR 3700 spectrophotometer was used to measure the transmittance of the crystals over the range of 250 nm to 900 nm. Structural characterisation was made by x-ray diffraction measurements on crushed crystals using a Rigaku SmartLab with a 9 kW rotating anode providing Cu Kα radiation. This arrangement employs a vertical goniometer and measured under a continuous scanning rate at 4 degrees per minute at 0.02° intervals of a 10° to 120° (2θ) range. HAXPES measurements were carried out at the I09 beamline at Diamond Light Source Ltd, using a photon energy of 5.91 keV with a semi-grazing angle between the beam and sample of 11°. The binding energy scale and the experimental resolution of 250 meV were determined from Au foil in electrical contact with the sample. The spectra were aligned to adventitious carbon, and to the intrinsic Fermi level of the doped sample, which were in excellent agreement. Core level peaks were normalised with respect to the Zn $2p$ maximum intensity, while the valence bands were normalised to the area under the curve.

References

(1) Dixon, S. C.; Sathasivam, S.; Williamson, B. A. D.; Scanlon, D. O.; Carmalt, C. J.; Parkin, I. P. Transparent conducting n-type ZnO:Sc – synthesis, optoelectronic properties and theoretical insight. *Journal of Materials Chemistry C* **2017**, *5*, 7585–7597.

(2) Chatratin, I.; Sabino, F. P.; Reunchan, P.; Limpijumnong, S.; Varley, J. B.; de Walle, C. G. V.; Janotti, A. Role of point defects in the electrical and optical properties of In2O3. *Physical Review Materials* **2019**, *3*, 074604.

(3) Scanlon, D. O.; Watson, G. W. On the possibility of p-type SnO2. *Journal of Materials Chemistry* **2012**, *22*, 25236.

(4) Janotti, A.; de Walle, C. G. V. Native point defects in ZnO. *Physical Review B* **2007**, *76*, 165202.

(5) Nishiyama, S.; Hattori, T. Electrical Conductivity and Thermoelectricity of ZnSb2O6 and (Zn1-xMx) Sb2O6 (M=Co, Ni, Cu) Ceramics. *Journal of the Ceramic Society of Japan* **2000**, *108*, 435–438.

(6) Kikuchi, N.; Hosono, H.; Kawazoe, H.; Tanegashima, O.; Ota, I.; Kimura, Y. Carrier Generation in Wide-Gap Conductor, Zinc Antimonate. *Journal of the American Ceramic Society* **2005**, *88*, 2793–2797.

(7) Kresse, G.; Hafner, J. Ab initio molecular dynamics for liquid metals. *Physical Review B* **1993**, *47*, 558–561.

(8) Kresse, G.; Hafner, J. Ab initio molecular-dynamics simulation of the liquid-metal–amorphous-semiconductor transition in germanium. *Physical Review B* **1994**, *49*, 14251–14269.

(9) Kresse, G.; Furthmüller, J. Efficiency of ab-initio total energy calculations for metals

and semiconductors using a plane-wave basis set. *Computational Materials Science* **1996**, *6*, 15–50.

(10) Kresse, G.; Furthmüller, J. Efficient iterative schemes for ab initio total-energy calculations using a plane-wave basis set. *Physical Review B* **1996**, *54*, 11169–11186.

(11) Blöchl, P. E. Projector augmented-wave method. *Physical Review B* **1994**, *50*, 17953–17979.

(12) Kresse, G.; Joubert, D. From ultrasoft pseudopotentials to the projector augmented-wave method. *Physical Review B* **1999**, *59*, 1758–1775.

(13) Adamo, C.; Barone, V. Toward reliable density functional methods without adjustable parameters: The PBE0 model. *The Journal of Chemical Physics* **1999**, *110*, 6158–6170.

(14) Paier, J.; Hirschl, R.; Marsman, M.; Kresse, G. The Perdew–Burke–Ernzerhof exchange-correlation functional applied to the G2-1 test set using a plane-wave basis set. *The Journal of Chemical Physics* **2005**, *122*, 234102.

(15) Ganose, A. M.; Scanlon, D. O. Band gap and work function tailoring of SnO2 for improved transparent conducting ability in photovoltaics. *Journal of Materials Chemistry C* **2016**, *4*, 1467–1475.

(16) Williamson, B. A. D. et al. Resonant Ta Doping for Enhanced Mobility in Transparent Conducting SnO2. *Chemistry of Materials* **2020**, *32*, 1964–1973.

(17) Baroni, S.; Resta, R. Ab initiocalculation of the macroscopic dielectric constant in silicon. *Physical Review B* **1986**, *33*, 7017.

(18) Gajdoš, M.; Hummer, K.; Kresse, G.; Furthmüller, J.; Bechstedt, F. Linear optical properties in the projector-augmented wave methodology. *Physical Review B* **2006**, *73*, 045112.

(19) Perdew, J. P.; Ruzsinszky, A.; Csonka, G. I.; Vydrov, O. A.; Scuseria, G. E.; Constantin, L. A.; Zhou, X.; Burke, K. Restoring the density-gradient expansion for exchange in solids and surfaces. *Physical Review Letters* **2008**, *100*, 136406.

(20) Ganose, A. M.; Jackson, A. J.; Scanlon, D. O. sumo: Command-line tools for plotting and analysis of periodic ab initio calculations. *Journal of Open Source Software* **2018**, *3*, 717.

(21) van Schilfgaarde, M.; Kotani, T.; Faleev, S. Quasiparticle Self-ConsistentGWTheory. *Physical Review Letters* **2006**, *96*, 226402.

(22) Chantis, A. N.; van Schilfgaarde, M.; Kotani, T. Ab InitioPrediction of Conduction Band Spin Splitting in Zinc Blende Semiconductors. *Physical Review Letters* **2006**, *96*, 086405.

(23) Kotani, T.; van Schilfgaarde, M.; Faleev, S. V. Quasiparticle self-consistentGWmethod: A basis for the independent-particle approximation. *Physical Review B* **2007**, *76*, 165106.

(24) Svane, A.; Christensen, N. E.; Gorczyca, I.; van Schilfgaarde, M.; Chantis, A. N.; Kotani, T. Quasiparticle self-consistent GW theory of III-V nitride semiconductors: Bands, gap bowing, and effective masses. *Physical Review B* **2010**, *82*, 115102.

(25) Punya, A.; Lambrecht, W. R. L. Band offsets between ZnGeN2, GaN, ZnO, and ZnSnN2and their potential impact for solar cells. *Physical Review B* **2013**, *88*, 075302.

(26) Buckeridge, J.; Scanlon, D.; Walsh, A.; Catlow, C. Automated procedure to determine the thermodynamic stability of a material and the range of chemical potentials necessary for its formation relative to competing phases and compounds. *Computer Physics Communications* **2014**, *185*, 330 – 338.

(27) Lany, S.; Zunger, A. Assessment of correction methods for the band-gap problem and for finite-size effects in supercell defect calculations: Case studies for ZnO and GaAs. *Physical Review B* **2008**, *78*, 235104.

(28) Lany, S.; Zunger, A. Accurate prediction of defect properties in density functional supercell calculations. *Modelling and Simulation in Materials Science and Engineering* **2009**, *17*, 084002.

(29) Makov, G.; Payne, M. C. Periodic boundary conditions in ab initio calculations. *Physical Review B* **1995**, *51*, 4014–4022.

(30) Murphy, S. T.; Hine, N. D. M. Anisotropic charge screening and supercell size convergence of defect formation energies. *Physical Review B* **2013**, *87*, 094111.

(31) Ganose, A. M.; Park, J.; Faghaninia, A.; Woods-Robinson, R.; Persson, K. A.; Jain, A. Efficient calculation of carrier scattering rates from first principles. *Nature Communications* **2021**, *12*, 2222.

(32) Poncé, S.; Li, W.; Reichardt, S.; Giustino, F. First-principles calculations of charge carrier mobility and conductivity in bulk semiconductors and two-dimensional materials. *Reports on Progress in Physics* **2020**, *83*, 036501.

(33) Buckeridge, J. Equilibrium point defect and charge carrier concentrations in a material determined through calculation of the self-consistent Fermi energy. *Computer Physics Communications* **2019**, *244*, 329–342.

(34) Wei, S.-H.; Zunger, A. Calculated natural band offsets of all II–VI and III–V semiconductors: Chemical trends and the role of cation d orbitals. *Applied Physics Letters* **1998**, *72*, 2011–2013.

(35) Brlec, K.; Davies, D. W.; Scanlon, D. O. Surfaxe: Systematic surface calculations. *Journal of Open Source Software* **2021**, *6*, 3171.

(36) Batzill, M.; Katsiev, K.; Burst, J. M.; Diebold, U.; Chaka, A. M.; Delley, B. Gas-phase-dependent properties ofSnO2(110), (100), and (101) single-crystal surfaces: Structure, composition, and electronic properties. *Physical Review B* **2005**, *72*, 165414.

(37) Mycielski, A. et al. The chemical vapour transport growth of ZnO single crystals. *Journal of Alloys and Compounds* **2004**, *371*, 150–152.

(38) Juskowiak, H.; Pajaczkowska, A. Chemical transport of β-Ga2O3 using chlorine as a transporting agent. *Journal of Materials Science* **1986**, *21*, 3430–3434.

(39) Ferreira, S. O., Ed. *Advanced Topics on Crystal Growth*; IntechOpen: London, 2013; Chapter Chemical Vapor Transport Reactions - Methods, Materials, Modeling.

(40) Legma, J.; Vacquier, G.; Casalot, A. Chemical vapour transport of molybdenum and tungsten diselenides by various transport agents. *Journal of Crystal Growth* **1993**, *130*, 253–258.
